# From Medical Records to AI-Ready Datasets: A Practical Guide for Clinical Researchers

**DOI:** 10.3390/jcm15166297

**Published:** 2026-08-14

**Authors:** Catalin Anghel, Andreea Alexandra Anghel, Marian Viorel Craciun, Simona Moldovanu, Adina Cocu, Diana-Elena Vulpe, Calina Maier, Vasile Potop, Christiana Diana Maria Dragosloveanu, Constantin Adrian Andrei, Serban Dragosloveanu, Cristian Scheau

**Affiliations:** 1 Department of Computer Science and Information Technology, “Dunărea de Jos” University of Galati, Științei St. 2, 800146 Galati, Romania; catalin.anghel@ugal.ro (C.A.); aa481@student.ugal.ro (A.A.A.); marian.craciun@ugal.ro (M.V.C.); simona.moldovanu@ugal.ro (S.M.); adina.cocu@ugal.ro (A.C.); 2 Faculty of Medicine, The “Carol Davila” University of Medicine and Pharmacy, 050474 Bucharest, Romania; diana-elena.plescan@drd.umfcd.ro (D.-E.V.); calina.maier@umfcd.ro (C.M.); andrei.constantinadrian@gmail.com (C.A.A.); cristian.scheau@umfcd.ro (C.S.); 3 “Foisor” Clinical Hospital of Orthopaedics, Traumatology and Osteoarticular TB, 021382 Bucharest, Romania; 4 Panait Sirbu Obstetrics and Gynaecology Hospital Bucharest, 060251 Bucharest, Romania; 5 Faculty of Dentistry, The “Carol Davila” University of Medicine and Pharmacy, 020021 Bucharest, Romania; christiana.dragosloveanu@umfcd.ro; 6 “Prof. Dr. Mircea Olteanu” Clinical Institute for Ophthalmological Emergencies, 010464 Bucharest, Romania

**Keywords:** clinical AI-readiness, medical datasets, machine learning, data collection, minimum common dataset, missing data, NaN values, data dictionary, medical imaging, clinical prediction

## Abstract

**Background**: Medical artificial intelligence (AI), machine learning (ML), and deep learning (DL) studies frequently begin with datasets collected for routine care rather than for computational modeling. Such datasets may contain inconsistent variables, heterogeneous measurement time points, unexplained NaN values, poorly defined outcomes, missing metadata, and insufficient documentation, which can compromise model development before any algorithm is selected. **Methods**: This Technical Note proposes a physician-facing Clinical AI-Readiness Guide for preparing medical datasets before AI-based analysis. The guide was developed as a practical framework organized around pre-modeling decisions, including the clinical task, cohort, minimum common dataset, outcome definition, predictor variables, measurement timing, missing-data logic, standardization, non-tabular data linkage, data dictionary, and validation readiness. **Results**: The proposed guide translates AI-readiness principles into concrete data-collection rules for clinical, laboratory, imaging, physiological-signal, textual, follow-up, and multimodal data. It emphasizes clinically consistent data acquisition, reliable target labeling, explicit missing-data logic, patient-level linkage, structured metadata, and validation feasibility. A structured checklist and scoring approach are also proposed as practical pre-modeling assessment tools to classify datasets as not ready, exploratory only, ML-ready with limitations, or AI-ready for model development. **Conclusions**: Medical AI-readiness should be established before model development begins. By helping physicians collect, structure, and document data more consistently, the proposed guide may improve collaboration between clinical and technical teams and reduce preventable dataset-related failures in medical AI research.

## 1. Introduction

Artificial intelligence (AI) and its subfields, including machine learning (ML) and deep learning (DL), are increasingly used in medicine for diagnostic support, risk prediction, prognosis estimation, image interpretation, treatment response assessment, workflow optimization, and clinical decision support [1,2]. These applications may use many forms of medical data, including structured clinical records, laboratory measurements, imaging examinations, physiological signals, pathology reports, free-text clinical documentation, longitudinal follow-up data, and multimodal combinations of these sources. Although discussions about medical AI often focus on model architecture, algorithm selection, and performance metrics, the validity of any AI-based study depends first on the clinical and methodological quality of the dataset used to train, test, and interpret the model.

This distinction is important because medical AI does not operate on abstract data. It operates on patient-level information that has been collected through clinical decisions, institutional workflows, diagnostic pathways, measurement protocols, and documentation practices. A laboratory value may be missing because the test was not clinically indicated, because the patient was less severe, because resources were limited, or because the test was performed in another institution. An imaging examination may differ across patients because scanners, protocols, acquisition parameters, or reporting practices are not standardized. A diagnostic label may be reliable when it is based on histopathology, expert consensus, or predefined criteria, but much weaker when it is inferred from an administrative code or from an imprecise clinical note. Therefore, the quality of a medical AI dataset is not only a technical property; it is also a clinical, procedural, and documentation property.

The concept of AI-ready health data has been increasingly recognized in recent literature. A qualitative study involving dataset experts showed that AI-readiness involves more than file format or computational accessibility; it also includes accuracy, completeness, consistency, ethical acquisition, fitness for a specific ML task, documentation, quality standards, and team-based dataset creation practices [3]. More recently, work on preparing clinical research data for AI-readiness in the context of the U.S. National Institute of Diabetes and Digestive and Kidney Diseases Data Centric Challenge presented a structured approach for transforming raw clinical research data into datasets suitable for downstream AI/ML applications [4]. Related recent work has also addressed explicitly AI-ready biomedical data resources [5], consolidated frameworks for assessing the quality of health data for secondary use [6], and multimodal AI approaches integrating imaging with clinical metadata [7]. These studies show that the dataset itself should be treated as a central methodological object, not merely as an input file delivered to the algorithm.

In everyday clinical research practice, however, many AI projects begin after data have already been collected. Physicians and clinical teams may therefore approach data scientists with retrospective datasets assembled from routine care that remain unsuitable for modeling because of inconsistent variables, unclear cohort or outcome definitions, heterogeneous measurement timing, incomplete follow-up, or undocumented missingness [3,4]. When such problems are discovered only after data extraction, the project may already be limited by decisions that can no longer be corrected.

Missing data are one of the most common and misunderstood problems in medical AI studies. In clinical datasets, missingness may reflect clinical indication, disease severity, follow-up processes, or data availability rather than random absence, and these mechanisms should be understood and documented before modeling [8]. For physicians planning AI studies, this means that missing data should be anticipated during study design, not treated only as a preprocessing inconvenience.

Another frequent source of invalid AI results is the lack of a clear temporal structure. In clinical practice, information appears over time: symptoms are recorded before diagnosis, laboratory tests may be repeated during hospitalization, treatment begins after clinical assessment, complications occur later, and outcomes may be measured at discharge, 30 days, 6 months, or 1 year. If a model uses variables that would not be available at the intended prediction moment, its apparent performance may be artificially high. This is a form of data leakage, a well-documented source of overoptimistic results and reproducibility failures in machine learning-based science [9]. For this reason, AI-ready medical datasets must specify not only what variables are present, but also when they become available in relation to the clinical decision or outcome being modeled.

These problems cannot be solved only by choosing a stronger algorithm. A technically analyzable dataset may still be clinically unsuitable if essential information was not collected, variables or outcomes are poorly defined, or their timing and meaning are insufficiently documented. The central question is not simply whether data exist, but whether the data are sufficiently complete, structured, documented, and clinically meaningful for the specific diagnostic, prognostic, monitoring, therapeutic, or operational question being asked.

The physician’s role in this preparation process is essential. Clinicians are responsible for defining the clinical problem, target population, outcome, relevant variables, measurement requirements, and follow-up logic that determine the meaning of the dataset. Data scientists can support database structuring, preprocessing, feature engineering, model development, and validation, but they cannot replace the clinical reasoning required to define the dataset. Clinical AI-readiness is therefore a collaborative responsibility, but the clinical definition of the dataset must remain clinician-led.

Several reporting guidelines and checklist-based standards developed by international expert groups have improved the transparency and appraisal of medical AI studies. TRIPOD+AI provides updated guidance for reporting studies that develop or evaluate clinical prediction models using regression or machine learning methods [10]. SPIRIT-AI and CONSORT-AI provide reporting guidance for clinical trial protocols and reports involving AI interventions [11]. CLAIM supports reporting of AI research in medical imaging [12], while STARD-AI provides reporting guidance for diagnostic accuracy studies using AI [13]. These guidelines are important because they help authors report what was done and help readers evaluate the credibility of completed or planned studies.

However, reporting guidelines do not fully address the earlier and practical problem faced by many clinical researchers: what should be collected before the study begins or before retrospective data extraction is performed? A physician planning an AI study must decide how to define the cohort, how to specify the outcome, which variables are mandatory, which variables are optional, how laboratory units will be standardized, how imaging or signal data will be linked to clinical records, how missing values will be documented, and how follow-up will be captured. These decisions are often made before a statistician or data scientist becomes involved. If they are made informally, the resulting dataset may be unsuitable for the intended AI task even if the final manuscript later follows a reporting checklist.

This Technical Note addresses that pre-modeling gap. Its focus is not the reporting of an already completed AI study and not the selection of a specific ML or DL algorithm. Instead, it provides a practical guide for preparing medical datasets before model development begins. The proposed approach is intended for physicians, clinical researchers, doctoral students, and multidisciplinary medical teams who plan to collect, extract, structure, or document data for AI-based studies. It is designed to accommodate tabular, laboratory, imaging, physiological-signal, textual, longitudinal, and multimodal clinical data.

The aim of this Technical Note is to propose a physician-facing Clinical AI-Readiness Guide for moving from routine medical records to AI-ready datasets. The guide focuses on the practical clinical and documentation decisions required to define the task, cohort, outcome, predictor set, measurement timing, missing-data logic, and dataset structure before modeling begins.

The purpose is to make the dataset preparation process explicit, reproducible, and understandable for both clinicians and data scientists.

The contribution of this Technical Note is to translate general data-readiness principles into a structured, physician-facing preparation layer that supports practical decisions during clinical data collection and extraction before model development. By doing so, the guide aims to improve collaboration between clinicians and technical teams and to reduce the number of medical AI projects compromised by preventable data collection and documentation problems.

The proposed framework is organized as a sequence of connected components. It progresses from pre-modeling decision points to practical decision blocks and data-acquisition rules, followed by a checklist, scoring approach, and blocking criteria that support the final readiness decision. This structure is intended to guide the reader from the conceptual basis of AI-readiness to its practical use before modeling begins.

**Figure 1:**
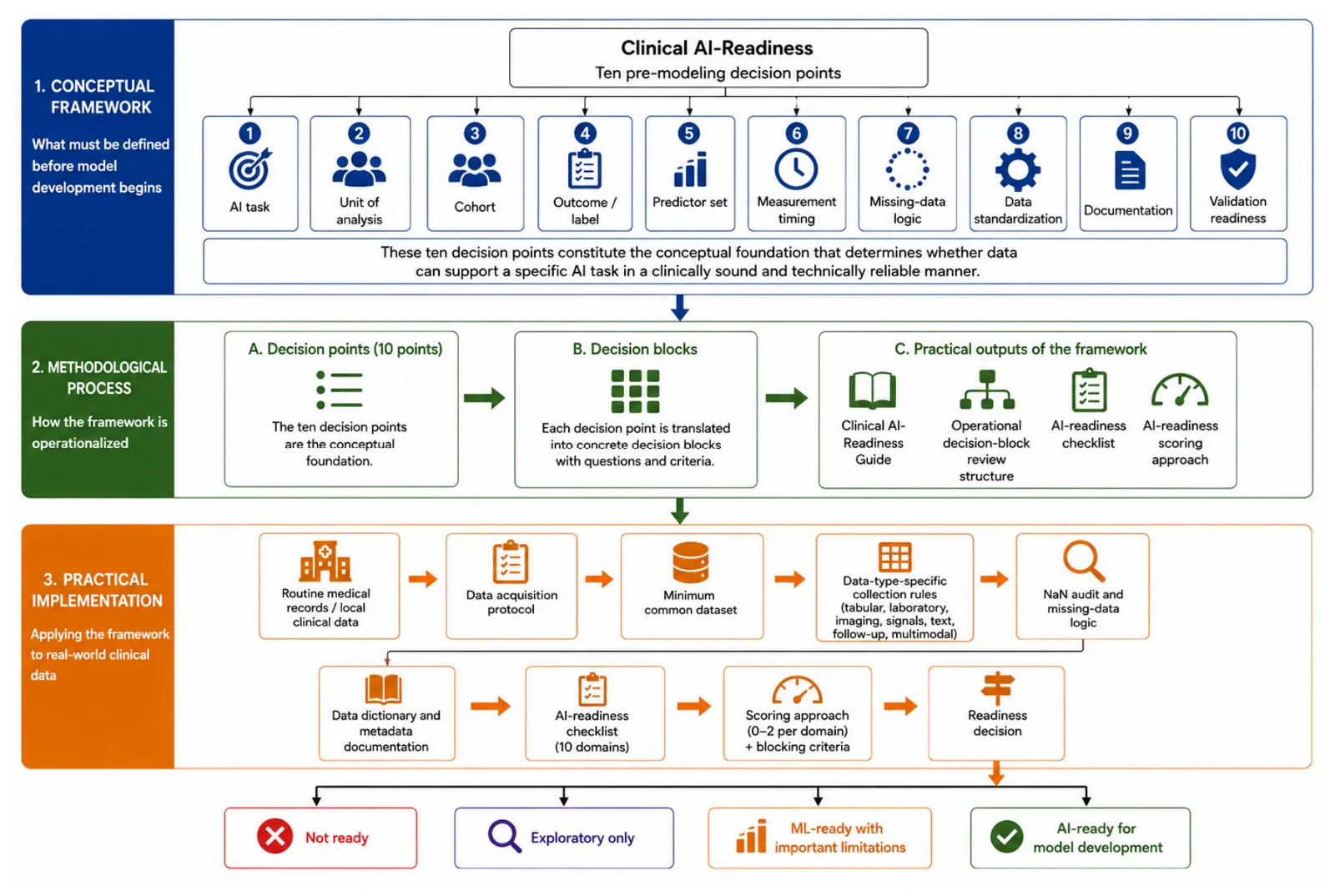
Conceptual framework, methodological process, and practical implementation of the Clinical AI-Readiness workflow.

Figure 1 provides a visual overview of this workflow, distinguishing the conceptual framework, methodological process, and practical implementation that connect the pre-modeling decision points to the final AI-readiness decision.

The workflow is organized into three connected levels. At the conceptual level, the ten pre-modeling decision points define the basis of Clinical AI-Readiness. At the methodological level, these decision points are translated into practical decision blocks and operational outputs. At the practical implementation level, the workflow starts from routine medical records or local clinical data and moves through the main preparation stages: data acquisition protocol, minimum common dataset, data-type-specific collection rules, NaN audit, and data dictionary preparation. These elements are then translated into the AI-readiness checklist, scoring approach, and blocking criteria, which together support the final readiness decision: not ready, exploratory only, ML-ready with important limitations, or AI-ready for model development.

## 2. Methodology

This Technical Note uses a framework development methodology. Its purpose is to define how the proposed Clinical AI-Readiness Guide was constructed, how its decision blocks were selected, and how these blocks are converted into practical outputs for clinical researchers. The methodology does not involve patient recruitment, clinical intervention, model training, or comparative algorithm evaluation. Instead, it formalizes a pre-modeling preparation procedure for medical datasets intended for artificial intelligence (AI), machine learning (ML), or deep learning (DL) studies.

The methodological object of this Technical Note is the dataset before modeling. In this context, the dataset is not treated only as a table, image archive, signal repository, or exported file. It is treated as a structured clinical object that must connect the intended medical question, the target population, the outcome definition, the predictor variables, the measurement timing, the documentation strategy, and the validation context. The guide was therefore constructed to help clinical researchers move from routine medical records or local data collections toward datasets that can be responsibly assessed for AI-based analysis.

### 2.1. Methodological Positioning

The proposed guide is positioned as a pre-modeling preparation framework. It is intended to be used before AI model development, either during prospective data collection planning or during retrospective data extraction from existing clinical sources. This positioning is important because many dataset weaknesses are determined before any algorithm is selected. Once the clinical question, cohort, outcome, variables, and measurement timing have been poorly defined, subsequent preprocessing can only partially reduce the problem.

The guide is not a reporting checklist for completed AI studies and does not replace established reporting standards. Its function is different: it translates dataset-level AI-readiness requirements into practical decisions that can be made by physicians and clinical teams before modeling begins. Recent work on data quality in AI has emphasized that reliable AI use depends on governance, ethics, documentation, FAIR principles, and fitness of data for intended computational use [14]. In the present Technical Note, these general principles are translated into clinical research decisions that can be applied at the dataset design and extraction stage.

The guide also differs from infrastructure-oriented approaches. Clinical data warehouses, data lakes, and data lakehouses can support large-scale healthcare AI by improving data integration, governance, scalability, metadata management, and analytics capability [15]. However, many physician-led AI studies begin at a smaller scale, with spreadsheets, registries, electronic record exports, imaging folders, laboratory files, or locally curated datasets. For these projects, the immediate methodological problem is not always institutional infrastructure, but whether the clinical dataset itself has been defined, structured, and documented in a way that supports the intended AI task.

### 2.2. Construction of the Clinical AI-Readiness Guide

The Clinical AI-Readiness Guide was constructed by decomposing dataset preparation into sequential decision points. The framework was developed as a literature-informed and expert-informed proposal rather than through a systematic review, Delphi procedure, or formal consensus methodology, and it was not adapted directly from a single existing framework. The ten decision points were identified by integrating recurring pre-modeling requirements described across the medical AI, clinical data-quality, dataset-readiness, and reporting literature with the clinical and technical expertise represented within the multidisciplinary author team. These decision points were selected because each one determines whether a medical dataset can be interpreted correctly before model development and because failure to define or document the corresponding domain can affect clinical interpretability, internal consistency, temporal validity, traceability, or the feasibility of subsequent model evaluation. The resulting structure follows the order in which clinical researchers should normally define a study: intended AI task, unit of analysis, cohort, outcome or label, predictor set, measurement timing, missing-data logic, data standardization, documentation, and validation readiness. The framework should therefore be regarded as a structured, expert-informed proposal grounded in existing literature and multidisciplinary practical expertise rather than as a consensus-derived or empirically validated instrument.

The first decision point is the AI task. The guide requires the intended task to be defined before variables are selected. A dataset prepared for diagnostic classification is not necessarily suitable for prognosis, treatment-response modeling, disease-severity assessment, monitoring, or workflow prediction. Therefore, the guide does not ask only whether data are available. It asks what the data are expected to support.

The second decision point is the unit of analysis. Medical data may be organized at the level of the patient, admission, clinical visit, lesion, image, signal segment, procedure, sample, or episode of care. This decision must be explicit because it affects sample size, independence of observations, train–test splitting, leakage risk, and interpretation of model outputs. For example, treating multiple admissions from the same patient as independent records may inflate the apparent amount of data if patient-level dependence is ignored.

The third decision point is the cohort. The guide requires inclusion criteria, exclusion criteria, clinical setting, study period, data source, and handling of repeated encounters to be defined before extraction or collection. This step determines the clinical population represented by the dataset. It also helps identify whether the dataset reflects the intended deployment context or only a narrow institutional subgroup.

The fourth decision point is the outcome or label. The outcome must be defined in clinical terms before modeling. The guide requires documentation of the outcome name, clinical definition, reference source, confirmation procedure, measurement time point, label type, and labeling authority. For diagnostic and classification tasks, the label should be connected to a defensible reference standard whenever possible. In imaging-based AI, recent recommendations for benchmark dataset creation also emphasize curation, representativeness, label quality, disease spectrum, acquisition conditions, and transparent annotation procedures as prerequisites for reproducible AI research [16]. For prognostic tasks, the follow-up interval and event definition must be explicit. For severity or treatment response tasks, the scoring system or clinical criteria must be documented before analysis.

The fifth decision point is the predictor set. Variables are separated into mandatory, recommended, and optional groups. Mandatory variables are those without which the dataset cannot support the main AI task. Recommended variables improve clinical interpretability, modeling quality, or adjustment for important confounders. Optional variables can support exploratory analyses but should not determine whether the dataset is usable. This classification is intended to prevent a common situation in which a dataset appears large and rich, while the variables needed for the primary clinical task are incomplete, inconsistently measured, or available only for selected patients.

The sixth decision point is measurement timing. Each candidate predictor must be assigned to a clinically meaningful time point, such as baseline, admission, diagnosis, pre-treatment, post-treatment, discharge, or follow-up. This timing rule determines whether a variable is eligible for the intended prediction or classification task. Variables recorded after the intended prediction moment should be excluded from predictor sets for earlier decisions. Recent evidence on same-admission prediction models has shown that diagnostic codes finalized after discharge can introduce label leakage when used as input features for outcomes that should be predicted during admission [17]. For this reason, the guide treats timing as a central AI-readiness requirement, not as a secondary annotation.

The seventh decision point is missing-data logic. The guide requires clinical teams to distinguish between different reasons for missingness. A value may be absent because it was not measured, not clinically indicated, unavailable in the source system, not extracted, not applicable to the patient, or lost during follow-up. These categories have different clinical meanings. Treating all missing values as equivalent can distort both model development and interpretation.

The eighth decision point is data standardization. The guide requires harmonization of measurement units, categorical coding, date formats, diagnostic terminology, medication names, imaging metadata, signal parameters, laboratory values, and follow-up event definitions. This step is included before modeling because standardization affects not only preprocessing but also clinical interpretation and reproducibility. Recent healthcare data quality work has emphasized accuracy, completeness, and reusability as key properties that influence downstream ML usability [18].

The ninth decision point is documentation. The guide requires a data dictionary before model development. The data dictionary should define each variable in clinical and technical terms, including variable name, definition, type, unit, allowed values, source, collection time point, missing-data rule, and role in the study. Recent work on medical dataset documentation and validation before ML analysis has proposed structured datasheets, checklists, data dictionaries, and validation tools, supporting the idea that documentation should be treated as a pre-modeling requirement rather than a final administrative step [19].

The tenth decision point is validation readiness. Before modeling, the dataset should be reviewed for sample size, class imbalance, outcome reliability, missingness, temporal consistency, leakage risk, and feasibility of internal or external validation. This review does not guarantee model performance. It determines whether the dataset is sufficiently prepared to justify model development or whether it should remain exploratory until additional data, better labels, or stronger documentation are available.

**Figure 2:**
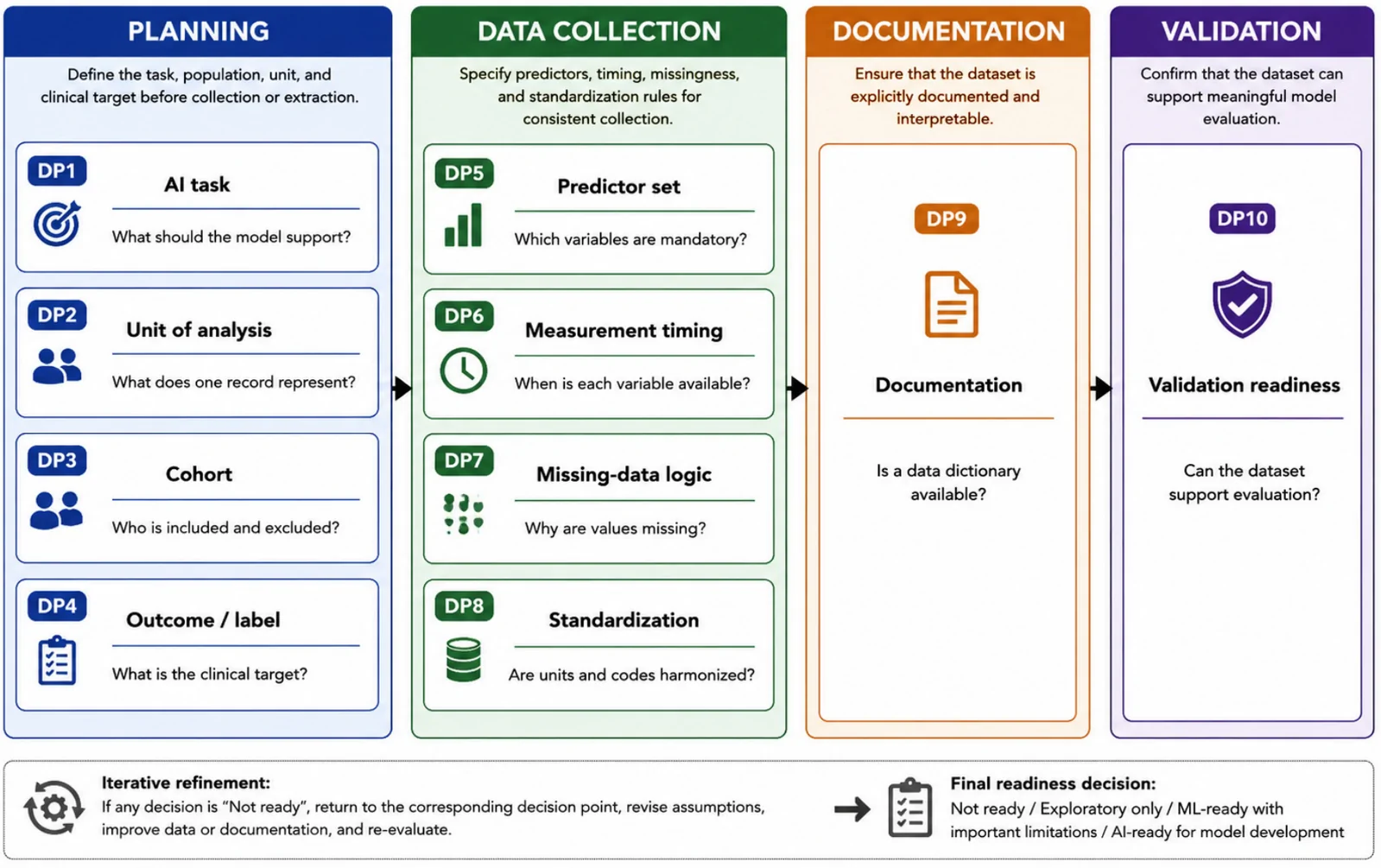
Ten pre-modeling decision points for clinical AI-readiness, grouped into Planning, Data Collection, Documentation, and Validation phases.

The ten decision points are summarized in Figure 2, where they are grouped into four broader phases: Planning, Data Collection, Documentation, and Validation. It presents the pre-modeling logic that clinical teams should clarify before data collection, retrospective extraction, or transfer to a technical team.

The process begins with the intended AI task and the unit of analysis, then moves through cohort definition, outcome or label specification, predictor planning, timing, missing-data logic, standardization, documentation, and validation readiness, organized into the broader phases of Planning, Data Collection, Documentation, and Validation.

### 2.3. Organization of the Guide into Decision Blocks

To make the decision points usable in practice, the guide converts them into decision blocks. Each block groups a core preparation requirement into a practical review unit that can be applied during data collection, retrospective extraction, or transfer to a technical team. This organization was chosen because physicians planning AI studies need actionable questions rather than generic statements about data quality.

The first block is clinical task definition. It requires the researcher to state what the future model is expected to do, in which clinical context, and at what decision point. The block also requires the researcher to define whether the intended use is diagnostic, prognostic, monitoring-oriented, treatment-related, severity-based, operational, or exploratory.

The second block is cohort definition. It requires the researcher to define the target population, data source, inclusion criteria, exclusion criteria, observation period, care setting, and unit of analysis. It also requires explicit rules for repeated measurements, multiple visits, bilateral conditions, multiple lesions, readmissions, and repeated procedures.

The third block is outcome definition. It requires the outcome name, clinical definition, confirmation source, timing, label type, and labeling authority. For binary outcomes, the positive and negative classes must be defined. For multiclass outcomes, all classes must be mutually interpretable. For ordinal outcomes, the order and clinical meaning of levels must be stated. For time-to-event outcomes, the start point, event definition, censoring rule, and follow-up window must be documented.

The fourth block is predictor planning. Each predictor should be described by clinical meaning, data type, unit, source, measurement time point, and role in the study. The predictor list is then divided into mandatory, recommended, and optional variables. This classification is useful for prospective data collection because it tells the clinical team which variables must be collected consistently and which variables may support secondary analyses.

The fifth block is timing and leakage control. This block asks whether each variable is available before the intended prediction moment. It also asks whether any variable is derived from the outcome, recorded after the outcome, or finalized after the decision point. Variables that violate the timing rule are flagged as potential leakage risks.

The sixth block is missing-data planning. This block requires the researcher to define how missing values will be represented and interpreted. It also requires the researcher to document why key variables may be missing. For example, a missing inflammatory marker because the test was not clinically indicated has a different meaning from a missing value caused by data extraction failure.

The seventh block is standardization. This block requires common units, consistent categorical coding, harmonized terminology, uniform date formats, and standardized file or metadata conventions where applicable. For imaging, signals, pathology, and text, this block also requires documentation of acquisition protocols, file formats, preprocessing assumptions, and linkage to patient-level data.

The eighth block is documentation. This block requires a data dictionary and, where relevant, additional metadata sheets for imaging, signal, text, or pathology data. The documentation should allow another researcher to understand what each variable means, how it was collected, when it was collected, and how it can be used.

The ninth block is validation readiness. This block asks whether the dataset can support meaningful model evaluation. It includes sample size, class balance, label reliability, completeness, temporal separation, patient-level independence, internal validation feasibility, and external validation potential.

The tenth block is readiness classification. After the previous blocks are completed, the dataset can be classified into a practical readiness level: not ready, exploratory only, ML-ready with limitations, or AI-ready for model development. This classification is not intended to replace expert judgment. It is intended to make dataset weaknesses explicit before modeling resources are invested.

**Figure 3:**
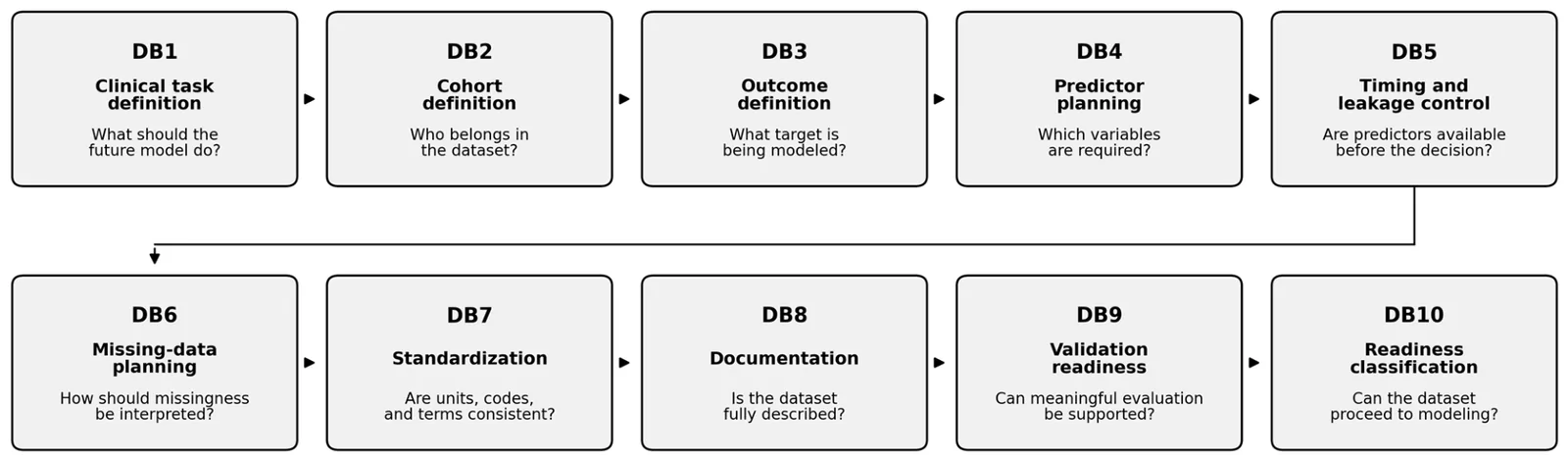
Decision-block structure of the Clinical AI-Readiness Guide.

The decision points described above are operationalized through a set of practical decision blocks. Figure 3 summarizes how these blocks translate the conceptual structure of AI-readiness into actionable questions for clinical teams.

The diagram emphasizes that the guide is not limited to a general assessment of data quality. Instead, it organizes dataset preparation into concrete blocks that can be reviewed before data collection, retrospective extraction, or transfer to a technical team. Each block addresses a practical requirement, from task and cohort definition to outcome specification, predictor planning, timing control, missing-data interpretation, standardization, documentation, validation readiness, and final readiness classification.

### 2.4. Practical Outputs of the Methodology

The methodology leads to a set of practical outputs intended to support clinical researchers during the preparation of medical datasets for AI-based studies. These outputs are designed to transform general AI-readiness principles into concrete decisions that can be applied before data collection, retrospective data extraction, or model development.

The first output is the Clinical AI-Readiness Guide itself. The guide organizes dataset preparation around the main decisions that determine whether medical data can support a specific AI task: clinical question, cohort, outcome, predictors, measurement timing, missing-data logic, standardization, documentation, and validation readiness. Its purpose is to help clinical teams move from routine medical records or locally assembled datasets toward data structures that are clinically interpretable and technically usable.

The second output is an operational decision-block review structure. This structure converts the guide into specific blocks that can be checked by the clinical team. Each block addresses a practical question: what is the intended clinical task, who is included in the cohort, what outcome is being predicted or classified, which variables are required, when are they measured, how missing values are interpreted, and whether the dataset can support meaningful validation.

The third output is an AI-readiness checklist. The checklist translates the guide into a practical verification tool that can be used before prospective data collection, before retrospective extraction, before data transfer to a technical team, or before model development. It is intended to make dataset weaknesses visible early, when they can still be corrected or documented.

The fourth output is an AI-readiness scoring approach. The score is designed as a screening tool for dataset maturity. It classifies the dataset according to its preparedness for AI analysis, ranging from not ready, to exploratory only, to ML-ready with limitations, and AI-ready for model development. The score does not certify model validity and does not replace clinical, statistical, or methodological review. Its role is to support structured decision-making before modeling begins.

Together, these outputs define the practical contribution of the proposed methodology: a physician-facing preparation layer that helps clinical researchers decide whether their data are sufficiently defined, complete, standardized, documented, and temporally appropriate for AI-based analysis.

## 3. Clinical AI-Readiness Guide for Medical Data Collection

This section presents the practical data-collection component of the Clinical AI-Readiness Guide. Its purpose is to help physicians and clinical researchers decide what medical data should be collected, when they should be collected, how they should be recorded, and how they should be documented before a dataset is transferred to a technical team or used for AI, ML, or DL model development. The section emphasizes that AI-ready data are not obtained by simply adding all available information to a spreadsheet. Instead, clinical data should be acquired according to a predefined protocol, using a minimum common dataset collected consistently across eligible patients, at clinically meaningful time points, with explicit rules for laboratory values, imaging examinations, physiological signals, textual records, outcomes, follow-up, missing values, and data dictionaries.

### 3.1. Establish the Data Acquisition Protocol Before Data Entry

The first practical step in preparing an AI-ready medical dataset is to establish a data acquisition protocol before any data are entered into a spreadsheet, exported from a hospital information system, collected from imaging archives, or transferred to a technical team. In many physician-led AI projects, data collection begins informally: patients are added as they are found, new columns are introduced during collection, laboratory tests are recorded only when available, and imaging or follow-up information is added inconsistently. This approach may create a large file, but it does not create a coherent dataset for AI, ML, or DL analysis.

The purpose of the data acquisition protocol is to ensure that all eligible cases are collected according to the same clinical and methodological logic. The protocol should define, before data entry begins, who is included, who is excluded, what one record represents, which variables are mandatory, when each variable is measured, how values are coded, how missing values are documented, and which source is used for each data element. This is particularly important in medical datasets because the same clinical variable may have different meanings depending on the patient group, measurement source, and time point of collection.

Table 1 summarizes the minimum elements that should be defined in a medical data acquisition protocol before the dataset is considered suitable for AI-oriented collection. The table is intended as a practical guide for clinicians and clinical researchers, showing not only what should be specified, but also the potential AI-related consequences of poor practice and how common poor practices can be converted into AI-ready data collection rules.

These elements should be treated as the minimum operational structure of the dataset. They do not replace the clinical research protocol, but translate it into concrete data-collection rules. Without these rules, the dataset may become heterogeneous in ways that are difficult or impossible to repair later. For example, if one clinician records laboratory values from admission and another records values from discharge, the resulting column no longer represents a single clinical variable. Similarly, if one group of patients has inflammatory markers and another group has renal markers, the dataset may contain many laboratory columns but lack a stable common basis for modeling.

The protocol should first define the patient group and the unit of analysis. The patient group is defined through the clinical condition, care setting, study period, inclusion criteria, and exclusion criteria. The unit of analysis defines what one row or one case in the dataset represents. Depending on the study, one record may correspond to one patient, one hospitalization, one outpatient visit, one surgical procedure, one lesion, one image, one signal segment, or one clinical episode. This decision must be explicit. For example, a dataset containing 500 radiographs from 100 patients is not equivalent to a dataset containing 500 independent patients. If several records come from the same patient, this must be documented from the beginning.

The protocol should then define the minimum data structure expected for each eligible case. For AI purposes, it is usually better to collect a smaller number of variables consistently than to create a large spreadsheet in which each patient has a different subset of information. A dataset in which half of the patients have CRP, ESR, fibrinogen, and leukocyte counts, while the other half have urea, creatinine, glucose, and liver enzymes, is not a coherent dataset for a single AI task unless this structure is intentional and clinically justified. If the main analysis depends on a specific group of variables, those variables should be collected for all eligible patients whenever possible.

The protocol must also define the clinical time point of each measurement. The same medical variable may have different meanings depending on when it is collected. A laboratory value at admission describes the patient’s baseline or presentation status. The same laboratory value after treatment may describe response to therapy. The same value at discharge may describe recovery, residual disease, or treatment effect. These values should not be merged into one column without a time-point definition. For example, “CRP” is not sufficiently precise. The dataset should distinguish variables such as CRP_at_admission, CRP_pre_treatment, CRP_post_treatment, CRP_at_discharge, or CRP_at_followup.

The same rule applies to imaging, physiological signals, textual records, procedures, and follow-up information. An initial CT scan, a post-treatment CT scan, and a follow-up CT scan do not represent the same clinical information. An ECG recorded at presentation and an ECG recorded after intervention should not be treated as interchangeable. A discharge letter contains information that was not available at admission and should not be used as an admission-time predictor. Therefore, the acquisition protocol should specify whether each data element is collected at baseline, admission, diagnosis, pre-treatment, post-treatment, discharge, or follow-up.

The protocol should classify variables as mandatory, recommended, or optional. Mandatory variables are required for the main AI task and should be collected for every eligible case. Recommended variables are clinically useful and should be collected when available, but the dataset may still be usable if some are missing. Optional variables are exploratory and should not determine whether a record can be included in the main analysis. This classification helps prevent the common situation in which a dataset appears rich because it has many columns, but the variables needed for the main task are missing or inconsistently collected.

For every variable, the protocol should identify the source of information. Clinical variables may come from admission notes, discharge letters, operative reports, laboratory information systems, imaging reports, device exports, pathology reports, or follow-up visits. If the same information can be found in more than one source, the protocol should define the preferred source. For example, a diagnosis confirmed by a specialist report may be preferable to an administrative code, and the date of surgery may be more reliable when taken from the operative report than from a billing field.

The protocol should also define how missing data are recorded. Empty cells should not be used as the only representation of missingness. A missing value may mean that the test was not performed, was not clinically indicated, was unavailable, was refused by the patient, was not applicable, was lost during follow-up, or was not extracted from the source system. These situations are different and should be documented differently when possible. Without this information, the technical team cannot distinguish between random missingness, clinically meaningful missingness, and extraction errors.

When a new variable is added after data collection has started, the clinical team should decide whether it can be recovered for all previously included cases. If it cannot, the variable should usually be treated as optional or exploratory rather than part of the main dataset. Similarly, if a variable is available at different time points for different patients, it should be split into separate time-point-specific variables rather than merged into a single mixed column.

Before data entry begins, the acquisition protocol should answer at least the following questions: Who is included? Who is excluded? What does one record represent? What outcome is being studied? Which variables are mandatory? At what clinical time point is each variable collected? What source is used for each variable? How are missing values documented? Who checks consistency during collection? If these questions cannot be answered, the dataset is not yet ready for AI-oriented data collection.

### 3.2. Define a Minimum Common Dataset for All Eligible Patients

After the data acquisition protocol is established, the clinical team should define a minimum common dataset. This is the smallest set of variables that must be collected for every eligible case included in the main analysis. The purpose of the minimum common dataset is to prevent the creation of a large but fragmented spreadsheet in which each patient has a different subset of clinical information.

For AI-based studies, a dataset with many columns is not necessarily a strong dataset. A smaller dataset with fewer variables collected consistently for all eligible patients is often more useful than a large dataset with many laboratory tests, imaging findings, or clinical variables available only for selected subgroups. The central question is not how many variables are present in the spreadsheet, but whether the variables required for the intended AI task are available, comparable, and clinically meaningful across the patients included in the analysis.

A common problem in medical datasets is irregular variable collection. For example, some patients may have inflammatory markers such as CRP, ESR, fibrinogen, and leukocyte count; other patients may have renal markers such as urea, creatinine, and uric acid; others may have liver enzymes, coagulation markers, or imaging data; and some may have only demographic information and diagnosis. Such a dataset may appear rich because it contains many variables, but it is weak for AI modeling if no stable core of variables is available for the same patient group.

The minimum common dataset should be defined before data entry begins. It should include only the variables that are essential for the main AI task and that can realistically be collected for most or all eligible patients. These variables should be clinically justified, clearly defined, measured at the same clinical time point, recorded using the same units and coding rules, and linked to a clearly defined outcome. Variables that are collected only in selected patients should be treated as recommended, optional, or exploratory unless the study is explicitly designed around that subgroup.

For example, in a study aiming to predict complications at 30 days after hospital admission, the minimum common dataset may include age, sex, main diagnosis, relevant comorbidities, admission laboratory markers, treatment category, and 30-day outcome. If CRP is available at admission for all patients, it can be part of the minimum common dataset. If procalcitonin was measured only in the most severe patients, it should not automatically be used as a mandatory predictor for the whole cohort. Its absence may reflect clinical severity or diagnostic suspicion rather than random missingness.

The same principle applies to imaging and other non-tabular data. If an imaging-based study includes CT scans, the minimum common dataset should not consist only of the image files. It should also include patient identifier, acquisition date, anatomical region, imaging protocol, relevant clinical label, and the relationship between the image and the outcome. If some patients have baseline imaging and others have follow-up imaging, these should not be treated as equivalent unless the study explicitly accounts for the difference.

Table 2 illustrates how the minimum common dataset can be adapted to different types of medical AI studies. The examples are not universal templates; they show the type of thinking required before data collection begins.

The minimum common dataset should be separated from the broader dataset. The broader dataset may contain additional variables that are useful for secondary analyses, subgroup exploration, or future hypotheses. However, the main AI task should rely on the minimum common dataset, not on an unstable collection of variables that differ from one patient to another. This distinction helps physicians understand that optional information can be valuable, but it cannot replace a consistent core dataset.

A practical rule is that every mandatory variable should answer three questions: Is it clinically relevant to the AI task? Can it be collected consistently for all eligible patients? Is it measured at the correct clinical time point? If the answer is no, the variable should not be part of the minimum common dataset. It may still be retained as optional, but it should not be required for the main model.

The minimum common dataset should also be reviewed during pilot data entry. Before collecting hundreds of cases, the clinical team should test the data structure on a small number of patients. This pilot check can reveal that some variables are not consistently available, that units are mixed, that time points are unclear, or that the outcome definition is difficult to apply. When such problems appear, the minimum common dataset should be revised before large-scale data collection continues.

In practical terms, the minimum common dataset is the foundation of AI-readiness. It ensures that eligible patients are described by the same essential information, collected in the same way, at the same relevant time point, and linked to the same type of outcome. Without this common core, the dataset may remain useful for descriptive clinical review, but it will be weak for reliable AI modeling.

### 3.3. Record Each Variable at a Consistent Clinical Time Point

After defining the minimum common dataset, the clinical team must specify the clinical time point at which each variable is collected. In medical AI datasets, a variable is not defined only by its name. It is defined by its name, unit, source, and measurement moment. A laboratory marker, imaging finding, clinical score, physiological signal, treatment variable, or follow-up event can have a different clinical meaning depending on whether it was recorded at admission, before diagnosis, before treatment, after treatment, at discharge, or during follow-up.

This is one of the most frequent reasons why medical datasets become difficult to use for AI analysis. A spreadsheet may contain a column named “CRP”, “creatinine”, “HbA1c”, “WBC”, “CT finding”, or “clinical score”, but if the values in that column were collected at different clinical moments for different patients, the column does not represent a single coherent variable. For some patients, the value may describe baseline status; for others, treatment response; for others, disease progression, complication, or recovery. Such mixed-time-point variables are difficult to interpret clinically and may weaken or invalidate model development.

The clinical time point should therefore be defined before data entry begins. For each variable, the data acquisition protocol should state whether the value is collected at baseline, admission, diagnosis, pre-treatment, intraoperative stage, post-treatment, discharge, follow-up, or another predefined moment. If the same variable is needed at more than one time point, it should be recorded in separate columns rather than merged into one column. For example, “CRP” is not sufficiently precise. Depending on the study, the dataset may need variables such as CRP_at_admission, CRP_pre_treatment, CRP_post_treatment, CRP_at_discharge, or CRP_at_followup. These variables should not be treated as interchangeable.

The same rule applies to other laboratory values. Creatinine measured at admission may describe baseline renal function, while creatinine measured postoperatively may indicate perioperative renal change or complication. Leukocyte count before antibiotic therapy reflects the initial infectious or inflammatory status, while leukocyte count after therapy may reflect treatment response. HbA1c measured at the first consultation describes baseline long-term glycemic control, whereas HbA1c measured after follow-up reflects longitudinal treatment effect. In each case, the marker name is the same, but the clinical meaning is different because the measurement moment is different.

This principle also applies to imaging data. An initial radiograph, a pre-treatment CT scan, a postoperative MRI, and a follow-up ultrasound do not represent the same type of clinical information. If the dataset contains medical images, each image should be linked to its acquisition date, clinical context, anatomical region, imaging modality, and relationship to the outcome. A baseline CT scan may be suitable as a predictor, while a post-treatment CT scan may describe response or progression. These images should not be stored as if they were equivalent observations unless the study is explicitly designed to compare them as separate time-point data.

Physiological signals also require time-point documentation. An ECG recorded at emergency presentation, an ECG recorded after intervention, and an ECG recorded during follow-up may reflect different clinical states. The same applies to EEG, EMG, spirometry, Holter monitoring, or sensor-based recordings. The dataset should document not only the signal file, but also the clinical moment of acquisition, recording duration, device or acquisition conditions, and whether the signal was obtained before or after diagnosis, treatment, or outcome occurrence.

Textual clinical data require the same caution. Admission notes, operative reports, discharge letters, radiology reports, pathology reports, and follow-up notes are not temporally equivalent. A discharge letter often contains information that was not available at admission and should not be used as a predictor for a model intended to support admission-time decisions. If free-text reports are used for NLP or LLM-based analysis, the dataset should document the document type, date, source, and clinical moment represented by the text.

The time point must match the intended AI task. If the model is intended to predict risk at admission, only information available at or before admission should be used as predictors. Laboratory values, imaging results, discharge diagnoses, complications, treatment decisions, or follow-up events that occur later should not be included as admission-time predictors. Using information that would not be available at the intended decision moment can introduce data leakage and produce overoptimistic model performance. Recent clinical ML evidence shows that leakage can artificially increase model performance and create a misleading impression of clinical utility [20].

For prognostic studies, the distinction between baseline predictors and follow-up outcomes is essential. Baseline predictors are variables available at the starting point of prediction, whereas outcomes are events measured later. For example, a model predicting 30-day readmission should clearly define whether predictors are taken at admission, during hospitalization, or at discharge. The readmission event should then be recorded as a follow-up outcome, not mixed with the predictor set. A model predicting 12-month complications should define the baseline time point, the follow-up window, and the event definition before data collection begins.

For treatment-response studies, pre-treatment and post-treatment variables must be separated. Pre-treatment variables may be used to predict response, while post-treatment variables describe the response itself or the evolution after therapy. If post-treatment measurements are used as predictors of treatment response, the model may appear accurate because it is using information that already reflects the outcome. Therefore, each variable should be assigned to a time point before deciding whether it is a predictor, outcome, adjustment variable, or exploratory feature.

The data dictionary should include a time-point field for every variable. This field should specify whether the variable was collected at admission, baseline, diagnosis, pre-treatment, post-treatment, discharge, or follow-up. When repeated measurements exist, the protocol should define whether the dataset uses the first value, last value, maximum value, minimum value, mean value, or all repeated values as a longitudinal sequence. These choices must be documented because different summary rules lead to different clinical interpretations.

A practical rule is that a variable should not be entered into the dataset unless the clinical team can answer three questions: when was it measured, why was it measured, and what clinical state does it represent? If these questions cannot be answered, the variable may still be retained for exploratory review, but it should not be treated as a reliable predictor for the main AI task.

In practical terms, AI-ready data collection requires temporal consistency. The same variable should be collected at the same clinical time point for all eligible patients, or it should be split into separate time-point-specific variables. Mixing values from admission, discharge, treatment, and follow-up in the same column creates ambiguity and weakens the dataset. For medical AI, when a value was collected is as important as what value was collected.

### 3.4. Collect Laboratory Data with Units, Timing, and Repeated-Measurement Rules

Laboratory data are among the most frequently used predictors in medical AI studies, but they are also among the easiest to collect incorrectly. A laboratory marker should never be recorded only as a numerical value. For AI-ready data collection, each laboratory variable should include at least five elements: the marker name, the numerical value, the unit of measurement, the clinical time point, and the source or context of measurement. Without these elements, the value may be technically present in the spreadsheet but clinically ambiguous.

The first requirement is to define which laboratory tests are mandatory for the main AI task. These tests should be selected before data collection begins and should be collected consistently for all eligible patients whenever possible. For example, in an infection-related study, the mandatory laboratory set may include CRP, leukocyte count, neutrophil count, lymphocyte count, and selected renal or hepatic markers. In a metabolic study, the mandatory set may include glucose, HbA1c, lipid profile, creatinine, and other relevant markers. The exact list depends on the clinical question, but the principle is constant: the main laboratory predictors should not differ randomly from one patient to another.

The second requirement is to separate mandatory laboratory tests from optional or exploratory tests. Some markers are performed only in specific clinical situations. For example, procalcitonin may be measured mainly in patients with suspected severe infection, D-dimer may be ordered mainly when thromboembolic disease is suspected, and specialized immunological or genetic tests may be available only for selected subgroups. Such variables may be clinically valuable, but they should not automatically become mandatory predictors for the entire dataset. If they are included in the main model without considering why they are missing in many patients, the model may learn patterns of clinical testing rather than patterns of disease.

The third requirement is to record the unit of measurement for every laboratory value. The same laboratory marker may be reported in different units across hospitals, laboratories, devices, or exported data systems. Glucose may be reported in mg/dL or mmol/L; creatinine may appear in mg/dL or µmol/L; CRP may be reported in mg/L or mg/dL; hemoglobin may appear in g/dL or g/L. These values cannot be merged safely unless the units are known and standardized. Recent work on large-scale clinical laboratory data has shown that inconsistent unit representations hinder secondary use and that automated unit standardization and harmonization can substantially improve interoperability and reliability for analytics and machine learning [21].

The fourth requirement is to avoid mixing values collected at different clinical moments. A laboratory marker measured at admission, before treatment, after treatment, at discharge, or during follow-up should be recorded as separate variables if more than one time point is relevant. For example, CRP_at_admission, CRP_post_treatment, and CRP_at_discharge describe different clinical states. Similarly, creatinine_preoperative and creatinine_postoperative should not be merged into a single creatinine column if the study concerns perioperative risk or complications. The name of the marker alone is not sufficient; the time point is part of the variable definition.

The fifth requirement is to define how repeated measurements are handled. Many hospitalized patients have repeated laboratory tests during the same episode of care. Before data extraction, the clinical team should decide whether the dataset will record the first value, last value, maximum value, minimum value, mean value, clinically worst value, or all repeated values as a longitudinal sequence. Each option has a different meaning. The first value may represent baseline status, the maximum value may represent disease severity, the last value may represent discharge status, and the full sequence may represent disease evolution. These rules should be defined before modeling, not chosen after inspecting which version gives the best performance.

Laboratory values should also be checked for plausibility. Extreme values may represent true critical results, but they may also reflect unit errors, transcription errors, decimal errors, or incorrect mapping. For example, a glucose value may appear implausible if mmol/L and mg/dL are confused, or a creatinine value may become clinically impossible if the unit is missing or converted incorrectly. Recent work on real-world oncology data showed that checking the relationship between laboratory codes, units, and reasonable value ranges can substantially improve conformance, consistency, completeness, and provenance in laboratory datasets [22]. This supports the practical need to document units and perform plausibility checks before using laboratory variables in AI studies.

When several laboratories or devices contribute data, the acquisition protocol should record the laboratory source where relevant. Different laboratories may use different assays, reference intervals, calibration procedures, or reporting conventions. In small single-center studies, this may be less problematic, but in multicenter or multi-source datasets it can affect comparability. When possible, the dataset should include the laboratory source, reference interval, or at least a note indicating whether all results come from the same laboratory system.

Laboratory variables should be named in a way that prevents ambiguity. Generic names such as “CRP”, “WBC”, “creatinine”, or “glucose” are often insufficient. AI-ready variable names should include the marker and, when relevant, the time point or role in the study. Examples include CRP_at_admission, WBC_pre_treatment, creatinine_preoperative, glucose_baseline, HbA1c_first_visit, or fibrinogen_at_diagnosis. This naming convention helps both clinicians and data scientists understand what each column represents without relying on memory or informal explanations.

The data dictionary should include a dedicated entry for every laboratory variable. This entry should specify the full test name, short variable name, unit, allowed range or plausibility range, time point, source laboratory or system, whether the variable is mandatory or optional, how missing values are coded, and how repeated measurements are summarized. If a variable was transformed, converted, or harmonized, the rule should also be documented.

In practical terms, laboratory data become AI-ready only when they are collected consistently, measured at defined clinical time points, recorded with units, checked for plausibility, and documented in the data dictionary. A spreadsheet containing many laboratory columns is not automatically useful for AI. A smaller set of laboratory markers collected in the same way, at the same clinical moment, with standardized units and clear missing-data rules, is often much stronger than a large but irregular laboratory dataset.

### 3.5. Collect Imaging Data with Acquisition Metadata and Patient-Level Linkage

Medical imaging data should not be collected as isolated image files. For an imaging dataset to be useful in an AI study, each image must be connected to the patient, the clinical context, the acquisition protocol, the time point, and the outcome or label used in the study. A folder containing radiographs, CT scans, MRI images, ultrasound images, endoscopic images, thermographic images, microscopy images, or pathology slides is not, by itself, an AI-ready imaging dataset. The clinical and technical metadata surrounding each image are part of the dataset and should be collected together with the image files.

The first requirement is to define the imaging modality and clinical purpose. The dataset should specify whether the images are radiographs, CT, MRI, ultrasound, mammography, thermography, endoscopy, microscopy, pathology slides, or another imaging modality. It should also state why the image was acquired in relation to the clinical task. An image acquired for initial diagnosis, preoperative planning, treatment monitoring, postoperative assessment, or follow-up does not represent the same clinical situation. Therefore, modality and clinical context should be recorded together.

The second requirement is patient-level linkage. Each image should be linked to a unique study identifier and, when relevant, to a specific admission, visit, lesion, anatomical region, procedure, or clinical episode. This linkage must be consistent across the imaging folder, clinical table, laboratory data, and outcome file. Without patient-level linkage, images cannot be reliably connected to clinical predictors, labels, follow-up outcomes, or validation groups. If several images are available for the same patient, the dataset should document whether they are repeated images of the same lesion, different anatomical regions, different time points, or separate clinical episodes.

The third requirement is acquisition metadata. For each image, the dataset should document at least the acquisition date, imaging modality, anatomical region, acquisition protocol, device or scanner information where available, file format, and image quality status. Depending on the modality, additional parameters may be required. For CT or MRI, these may include contrast use, sequence, slice thickness, reconstruction protocol, field of view, and orientation. For ultrasound, relevant information may include probe type, view, operator-dependent acquisition conditions, and measurement plane. For thermography, acquisition distance, room temperature, acclimatization time, camera model, and region of interest are important. For endoscopy or microscopy, magnification, acquisition device, preparation method, and field selection may be relevant.

Acquisition parameters should be treated as meaningful information, not as secondary technical details. Recent evidence in screening mammography showed that acquisition parameters influenced the interpretive performance of both AI systems and radiologists [23]. Although this evidence comes from mammography, the practical implication is broader: image appearance and downstream AI behavior can be affected by the way the image was acquired. For this reason, acquisition metadata should be retained whenever possible, especially in multicenter, multi-device, or multi-protocol datasets.

The fourth requirement is time-point definition. Imaging data should be labeled according to when they were acquired in the clinical pathway. A baseline CT scan, a post-treatment CT scan, a postoperative radiograph, and a follow-up MRI should not be mixed as if they were equivalent images. If the AI task is diagnostic, the relevant imaging time point may be the examination performed before final diagnosis or before treatment. If the task is treatment-response assessment, both baseline and post-treatment images may be needed, but they should be stored as separate time-point-specific records. If the task is prognosis, imaging predictors should be restricted to images available at the defined prediction moment.

The fifth requirement is label definition and annotation transparency. Each image or image group should be linked to a clearly defined label or outcome. The label may come from radiologist interpretation, pathology confirmation, surgical findings, expert consensus, follow-up, clinical diagnosis, or another predefined reference source. The dataset should document who assigned the label, what criteria were used, and whether the label applies to the whole image, a region of interest, a lesion, a slice, a frame, or the patient as a whole. Recommendations for reproducible AI benchmark datasets in radiology emphasize the importance of dataset curation, representativeness, label quality, disease spectrum, acquisition conditions, and transparent annotation procedures [16]. These principles should also guide smaller physician-led imaging datasets, even when the dataset is not intended to become a public benchmark.

The sixth requirement is file organization and naming. Imaging files should be stored using a consistent structure that preserves the relationship between patient, modality, time point, and label. File names should not contain identifiable patient information, but they should allow linkage through a secure study identifier. A practical structure may separate files by modality, patient identifier, time point, and image type. When DICOM files are available, they should generally be preserved because they contain important technical metadata. If images are exported to JPEG, PNG, TIFF, NIfTI, or another format, the export rule should be documented, and the team should record whether metadata were retained, removed, or stored separately.

The seventh requirement is image quality control. Before modeling, the clinical team should define whether images with motion artifacts, incomplete anatomical coverage, incorrect positioning, poor exposure, low resolution, compression artifacts, missing sequences, or non-standard acquisition should be included or excluded. If such images are retained, the quality issue should be documented. The decision should be made before model development, not after performance is observed. A model trained on inconsistent or poor-quality images may learn acquisition artifacts, device-specific patterns, or documentation bias instead of clinically relevant findings.

The eighth requirement is privacy and de-identification. Medical images may contain identifiable information not only in metadata, but also in pixel-level image content. Recent work on privacy in medical imaging AI highlights risks related to both metadata and pixel-level information, including identifiers, acquisition details, institutional information, and image-intrinsic features that may support re-identification [24]. Therefore, imaging datasets should be reviewed for patient identifiers in DICOM headers, burned-in annotations, facial structures, institutional details, accession numbers, dates, and other potentially identifying elements. De-identification should be performed before data sharing, but it should not destroy the study identifiers needed for secure linkage between images, clinical variables, and outcomes.

The ninth requirement is separation of image-level and patient-level analysis. In imaging AI, the number of image files is often larger than the number of patients. This can create a false impression of sample size. A dataset with 5000 images from 200 patients is not equivalent to a dataset with 5000 independent patients. If multiple images from the same patient are split across training and testing sets, the model may be evaluated on data that are not truly independent. Therefore, the dataset should record patient-level identifiers and should allow later validation to be performed at the appropriate patient, lesion, or episode level.

A practical imaging data acquisition record should therefore include study identifier, patient identifier, modality, anatomical region, acquisition date, clinical time point, device or protocol information, file format, quality status, label source, annotation procedure, and linkage to the clinical outcome. These elements allow the technical team to understand not only what the image shows, but also how the image was obtained, when it was obtained, to whom it belongs, and what clinical question it supports.

In practical terms, imaging data become AI-ready when the images are not treated as detached files, but as clinically contextualized records. Each image should be linked to the correct patient, time point, acquisition protocol, label, and outcome. Without this context, even a large image folder may be insufficient for reliable AI development.

### 3.6. Collect Physiological Signals and Device-Based Data with Technical and Clinical Context

Physiological signals and device-based data require a dedicated acquisition plan because they are not defined only by the measured physiological phenomenon. They are also defined by the device, sensors, sampling frequency, recording duration, channel configuration, acquisition conditions, preprocessing assumptions, and clinical time point. ECG, EEG, EMG, spirometry, Holter monitoring, photoplethysmography, electrodermal activity, continuous glucose monitoring, wearable accelerometry, and other sensor-derived data can be valuable for AI studies, but they become difficult to interpret when the acquisition context is missing.

The first requirement is to define the type of signal and its clinical purpose. The dataset should specify whether the signal is used for diagnosis, monitoring, prognosis, treatment-response assessment, activity detection, disease-severity estimation, or continuous follow-up. For example, an ECG collected at emergency presentation for suspected myocardial infarction, a Holter recording used for arrhythmia monitoring, and an ECG recorded after intervention do not represent the same clinical context. Similarly, a wearable-derived signal collected during daily life is different from a signal acquired during a structured clinical test.

The second requirement is device and sensor documentation. For every signal source, the dataset should document the device name or type, sensor modality, number of channels, sampling frequency, recording duration, placement or anatomical location, and acquisition conditions. In wearable datasets, this may include accelerometry, photoplethysmography, electrodermal activity, skin temperature, blood volume pulse, heart rate, or heart rate variability. A recent Scientific Data descriptor of wearable physiological signals explicitly documents the wearable device, sensor modalities, sampling frequencies, structured stress and exercise protocols, and labeled phases of the recordings, illustrating the level of acquisition information needed for reusable physiological-signal datasets [25].

The third requirement is timing and clinical-state documentation. A physiological signal should be linked to the clinical moment at which it was recorded. ECG_at_presentation, ECG_post_intervention, EEG_during_event, EMG_pre_treatment, spirometry_baseline, and CGM_followup represent different types of information. If the study aims to predict an early clinical decision, signals recorded after the decision or after treatment should not be used as baseline predictors. If the study concerns monitoring or response, repeated signals may be appropriate, but the sequence and timing must be explicitly documented.

The fourth requirement is recording condition. Physiological signals are highly sensitive to acquisition context. The dataset should state whether the signal was recorded at rest, during exercise, during sleep, during stress induction, during a procedure, during routine monitoring, or during free-living conditions. For wearable or ambulatory recordings, the protocol should document activity context where possible, because motion, posture, exercise, stress, sleep, and environmental conditions can influence the signal. For clinical recordings, the protocol should document whether the recording was obtained before medication, after medication, before intervention, after intervention, or during a specific clinical episode.

The fifth requirement is segmentation. Many physiological signals are continuous or repeated over time. Before modeling, the clinical team should define whether the analysis uses the full recording, fixed-length segments, clinically defined episodes, event-centered windows, averaged features, peak values, or manually selected intervals. Segment selection should not be performed informally after viewing the data, because this may introduce bias or make the study difficult to reproduce. The segmentation rule should be defined in the data acquisition or preprocessing protocol and recorded in the data dictionary.

The sixth requirement is signal quality and artifact documentation. ECG, EEG, EMG, PPG, accelerometry, and wearable signals may contain noise, missing segments, motion artifacts, electrode displacement, poor contact, device interruption, or environmental interference. These issues should be labeled or documented before modeling. A signal segment with motion artifacts is not necessarily unusable, but the model and the evaluator must know that the artifact exists. In ECG-based deep learning research, recent evidence has emphasized challenges such as noise, interference, imbalanced datasets, limited standardization, limited external validation, and inconsistent reporting [26]. These issues reinforce the need to document acquisition and quality conditions before AI analysis.

The seventh requirement is synchronization with clinical data. Physiological signals often need to be linked to clinical variables, laboratory measurements, imaging findings, medication exposure, procedures, outcomes, or follow-up events. This linkage requires a common patient identifier and a clear time reference. For example, a Holter recording used to predict a cardiac event must be linked to the correct patient, recording date, medication status, and follow-up window. A continuous glucose monitoring sequence used for glycemic prediction must be linked to meal timing, insulin use, exercise, medication changes, or other relevant contextual variables when these are part of the intended AI task.

The eighth requirement is clarity regarding raw data and derived features. The dataset should specify whether it contains raw signals, preprocessed signals, extracted features, or both. For example, ECG data may be stored as raw waveforms, rhythm labels, intervals, or derived features. Wearable data may include raw accelerometry, heart rate, heart rate variability, electrodermal activity, or summary statistics. Derived features should be documented with their calculation method, time window, and preprocessing assumptions. If only derived features are stored, the loss of access to raw signal information should be acknowledged.

The ninth requirement is handling of device heterogeneity. In some studies, all signals are recorded using the same device and protocol. In others, signals may come from different ECG machines, EEG systems, spirometers, wearable devices, software versions, or hospital units. Device heterogeneity should be documented because models may learn device-specific patterns rather than physiological or clinical patterns. If multiple devices are used, the dataset should include the device type or source as metadata and should allow later assessment of device-related bias or generalizability.

The tenth requirement is privacy and governance. Physiological signals may not contain obvious identifiers, but they can still be sensitive. ECG, EEG, wearable, and continuous monitoring data are patient-level biomedical records and should be handled with appropriate governance, anonymization, access control, and linkage protection. When signals are combined with clinical records, laboratory data, imaging, or follow-up outcomes, the risk of re-identification may increase. Therefore, study identifiers should preserve secure linkage while avoiding direct patient identifiers in the analysis files.

The data dictionary should include a dedicated description for every physiological-signal variable or file. This description should include signal type, device, sensor, channel, sampling frequency, recording duration, clinical time point, acquisition condition, preprocessing status, segmentation rule, quality flag, patient linkage, and outcome linkage. For wearable or continuous monitoring datasets, it should also document whether the data were collected under controlled conditions, free-living conditions, exercise, sleep, stress, or routine clinical monitoring. Reviews of AI and wearable technology in diabetes management show that AI models are increasingly applied to physiological data from devices such as continuous glucose monitors, fitness trackers, and smartwatches, which makes documentation of sensor modality, clinical endpoint, and population context essential [27].

In practical terms, physiological signals become AI-ready when the waveform, recording, or sensor sequence is not treated as an isolated file. It must be linked to the patient, device, clinical state, acquisition conditions, time point, quality status, and outcome. Without this context, even large volumes of signal data may be unsuitable for reliable AI modeling.

### 3.7. Collect Textual Clinical Data with Source, Structure, Timing, and De-Identification Rules

Textual clinical data can be valuable in medical AI studies because they often contain information that is absent, incomplete, or less detailed in structured fields. Clinical notes, admission records, discharge letters, operative reports, radiology reports, pathology reports, consultation notes, nursing notes, and follow-up documents may include symptoms, clinical reasoning, diagnostic impressions, treatment decisions, social context, complications, and longitudinal evolution. However, free-text data are not AI-ready simply because they exist in the electronic health record or can be copied into a document file.

The first requirement is to define the type and source of each textual record. The dataset should specify whether the text comes from an admission note, progress note, discharge letter, radiology report, pathology report, operative report, emergency department note, follow-up note, or another clinical document. These document types are not interchangeable. An admission note describes the initial clinical presentation, while a discharge letter summarizes information accumulated during hospitalization. A radiology report represents imaging interpretation, while a pathology report may provide diagnostic confirmation. Therefore, the document source must be recorded as part of the dataset.

The second requirement is to document the clinical time point of the text. A clinical note written before diagnosis does not contain the same type of information as a discharge summary or a follow-up report. If an AI model is intended to support a decision at admission, text generated after hospitalization, treatment, or outcome occurrence should not be used as an admission-time predictor. Similarly, if a study uses pathology reports as labels, those reports should be separated from predictor text. The data acquisition protocol should therefore record the date, document type, and clinical moment represented by each textual record.

The third requirement is to decide whether textual data will be used as raw text, manually extracted variables, automatically extracted concepts, or supporting documentation for labels. These are different use cases. Raw text may be used for natural language processing or large language model-based analysis. Manually extracted variables may be entered into structured columns. Automatically extracted concepts may be used as features, labels, or supporting variables. Supporting documents may be used only to confirm outcomes or adjudicate labels. The intended role of the text should be defined before extraction.

The fourth requirement is to avoid mixing structured and unstructured information without documentation. Structured fields and free-text notes may contain overlapping but not identical information. A recent large-scale EHR study showed that structured and unstructured data can be complementary, with free-text notes containing important additional information that may not be reflected in structured fields [28]. For this reason, if the same clinical concept is available both as a structured code and in free text, the dataset should define which source is preferred, whether both are retained, and how discrepancies are resolved.

The fifth requirement is to preserve enough context for interpretation. Extracting isolated words or phrases from clinical text may remove important meaning. For example, “infection” may refer to suspected infection, ruled-out infection, previous infection, family history, postoperative infection, or confirmed infection. Similarly, “no evidence of malignancy” should not be reduced to “malignancy” without negation handling. If textual data are converted into structured variables, the extraction rule should account for negation, uncertainty, temporality, experiencer, and document context.

The sixth requirement is to document any information extraction process. If clinical concepts are extracted from free text using manual review, rule-based methods, natural language processing, or large language models, the dataset should document the extraction method, reviewer role, model or tool used, prompt or rule set where applicable, validation procedure, and error-checking strategy. Recent work has shown that open-source large language models can be used to extract social determinants of health from free-text electronic health records, but the extraction process still requires evaluation against manually labeled data and attention to hallucination or extraction errors [29]. Therefore, textual extraction should not be treated as automatically reliable without validation.

The seventh requirement is to define how dates and temporal expressions are handled. Clinical text often contains dates, durations, relative expressions, and temporal relationships, such as “two weeks before admission”, “postoperative day 3”, “after chemotherapy”, or “at 6-month follow-up”. These expressions may be essential for reconstructing the clinical timeline. If they are extracted or normalized, the method should be documented. Recent work on de-identification and temporal normalization of electronic health record notes shows that inconsistent time formats hinder interpretation and that date normalization is closely linked to the secondary use of clinical text [30].

The eighth requirement is de-identification. Textual clinical data often contain names, initials, dates, addresses, phone numbers, hospital numbers, physician names, locations, rare events, or contextual details that may identify the patient. De-identification should be performed before data sharing or technical processing outside the approved clinical environment. The de-identification procedure should document what categories of sensitive information were removed, replaced, generalized, or retained under secure study identifiers. At the same time, de-identification should preserve the clinical information needed for the study, such as the relationship between the text, the patient, the clinical event, and the outcome.

The ninth requirement is linkage to structured data and outcomes. A text document should be linked to the correct patient, encounter, time point, and outcome. If a radiology report is used, it should be linked to the corresponding image or examination. If a pathology report is used, it should be linked to the relevant specimen, procedure, diagnosis, and outcome. If a discharge summary is used, it should be linked to the hospitalization episode and follow-up window. Without this linkage, textual data may become clinically rich but analytically disconnected from the AI task.

The tenth requirement is quality control. The dataset should document whether text is complete, duplicated, truncated, copied from previous visits, automatically generated, templated, or manually written. Copy-forward text may preserve outdated information, templated reports may contain default phrases, and duplicated documents may inflate the apparent amount of data. These issues should be identified before textual data are used for AI modeling.

For AI-ready collection, each textual record should therefore include at least the following metadata: patient identifier, document type, document source, date, clinical time point, author role where relevant, de-identification status, intended use, linkage to structured variables, linkage to outcome or label, and extraction method if concepts are derived from text. If free text is used directly for modeling, the dataset should also document preprocessing steps such as tokenization, translation, spelling correction, removal of identifiers, section selection, or exclusion of irrelevant document parts.

In practical terms, textual clinical data become AI-ready when they are treated as dated, sourced, de-identified, and clinically contextualized documents. Copying notes into a file is not enough. The clinical team must know what type of document the text represents, when it was written, what clinical information it contains, how it was de-identified, whether it is used as a predictor or label source, and how it links to the rest of the dataset.

### 3.8. Collect Outcomes and Follow-Up Data Explicitly

An AI-ready medical dataset must include a clearly defined outcome. The outcome is the clinical result that the model is expected to predict, classify, detect, explain, or monitor. Without a reliable outcome, the dataset may contain many patients, many variables, and many measurements, but it will not support a valid AI task. For this reason, outcome and follow-up data should be planned before data collection begins, not reconstructed informally after the spreadsheet has already been completed.

The first requirement is to define the outcome in clinical terms. Labels such as “severe”, “complicated”, “improved”, “poor evolution”, “high risk”, or “treatment failure” are not sufficient unless they are linked to explicit criteria. A severity outcome, for example, may be defined by intensive care admission, mechanical ventilation, major complication, revision surgery, mortality, or a validated clinical score. A treatment-response outcome may be defined by symptom improvement, biomarker reduction, imaging response, functional score change, disease remission, or recurrence-free status. The dataset should specify the exact criteria used to assign each outcome label.

The second requirement is to define the source of outcome confirmation. An outcome may be confirmed by histopathology, imaging, laboratory criteria, specialist diagnosis, operative findings, validated clinical score, adjudication committee, discharge diagnosis, follow-up visit, registry data, mortality records, or patient-reported outcome measures. These sources do not have the same reliability. For example, a histopathology-confirmed diagnosis is different from a suspected diagnosis written in a clinical note, and a surgically confirmed complication is different from an administrative code. The outcome source should therefore be recorded in the dataset.

The third requirement is to define when the outcome is measured. A diagnostic outcome may be assessed at the time of diagnosis, while a prognostic outcome may occur days, months, or years later. A treatment-response outcome may be measured after a specific treatment interval. A postoperative complication may be assessed during hospitalization, at 30 days, at 90 days, or at 1 year. These are different outcomes. For example, in-hospital mortality, 30-day mortality, and 1-year mortality should not be merged without distinction. Similarly, infection during hospitalization and infection within 90 days after surgery may reflect different clinical processes and should be defined separately.

The fourth requirement is to separate predictors from outcomes. This is especially important in prognostic and treatment-response studies. Predictors are variables available at the defined prediction moment. Outcomes are events or states measured later. If the model is intended to predict 30-day readmission, the readmission event is an outcome and should not be included among baseline predictors. If the model is intended to predict treatment response, post-treatment variables that directly reflect response should not be used as predictors unless the task is explicitly monitoring rather than baseline prediction. Recent work on sequential electronic health record data shows that temporal patient information, visit timing, and outcome definition are central issues in outcome prediction models [31].

The fifth requirement is to define the follow-up window. Follow-up should not be described only as “available” or “not available”. The dataset should state whether follow-up is measured at discharge, 7 days, 30 days, 90 days, 6 months, 1 year, or another clinically justified interval. The follow-up window should be the same for all eligible patients whenever possible. If different follow-up durations are used, the dataset should document the reason and define how patients with shorter follow-up are handled.

The sixth requirement is to document censoring and loss to follow-up when relevant. In time-to-event or survival-type studies, some patients may not experience the event during the observation period, and some may be lost before the planned follow-up time. These patients should not simply be coded as having no event unless the follow-up window is complete. The dataset should distinguish between no event observed, event occurred, lost to follow-up, follow-up incomplete, and not applicable. Without this distinction, the model may learn from incorrectly labeled outcomes.

The seventh requirement is to define the label type. The outcome may be binary, multiclass, ordinal, continuous, time-to-event, or longitudinal. A binary label may distinguish disease present versus absent, complication versus no complication, or response versus no response. A multiclass label may distinguish disease subtypes. An ordinal label may represent severity stages. A continuous label may represent a score or biomarker value. A time-to-event label may require event date and censoring status. A longitudinal outcome may require repeated measurements across time. The label type determines how the dataset should be structured and how the model can be evaluated.

The eighth requirement is to document who assigns the outcome label. In some studies, the label may be assigned automatically from a structured field. In others, it may require manual review by one clinician, independent review by two clinicians, expert consensus, or adjudication. If the label is based on manual review, the reviewer role, specialty, number of reviewers, disagreement handling, and adjudication procedure should be documented. If the label is extracted from text or codes, the extraction rule should be recorded.

The ninth requirement is to avoid vague retrospective relabeling. When outcomes are assigned after looking at the available data, there is a risk that labels become inconsistent or influenced by the variables intended to be used as predictors. For example, if “high risk” is defined informally using the same laboratory markers that will later be used to train the model, the outcome is not independent of the predictors. The clinical criteria for outcome assignment should be defined before modeling and, ideally, before extraction.

The tenth requirement is to preserve the link between outcome, patient, and time. Each outcome should be linked to the correct patient identifier, clinical episode, measurement date, and follow-up window. If a patient has several admissions, procedures, lesions, or images, the outcome must be linked to the correct unit of analysis. For example, a postoperative infection must be linked to the relevant surgical procedure, not only to the patient in general. A lesion-level diagnosis must be linked to the corresponding lesion or image, not simply to the patient record.

For clinical-trial or interventional datasets, outcome collection may include adverse events, serious adverse events, mortality, patient dropout, treatment failure, efficacy outcomes, or trial completion outcomes. Recent AI-ready clinical trial datasets show that these outcome types require explicit task definitions and structured links between multimodal features and prediction targets [32]. The same principle applies to smaller clinical datasets: the outcome must be defined as a usable prediction target, not treated as an informal description of patient evolution.

A practical outcome record should therefore include: outcome name, clinical definition, label type, confirmation source, measurement date, follow-up window, event status, censoring or loss-to-follow-up status where relevant, reviewer or label source, and linkage to the correct patient or clinical episode. If these elements are missing, the dataset may be unsuitable for supervised AI modeling even if the predictor variables are well collected.

In practical terms, outcome and follow-up data are the anchor of the AI task. A dataset cannot be AI-ready if the target result is vague, inconsistently measured, or disconnected from the predictors. Physicians should therefore define the outcome with the same care as they define the predictors. The model can only learn a clinically meaningful pattern if the clinical result it is asked to predict is itself clearly defined, consistently measured, and properly linked to the patient timeline.

### 3.9. Document and Resolve NaN Values Before Modeling

Missing values are one of the most damaging problems in physician-prepared datasets for AI and ML studies. In practice, they usually appear as empty cells, “NA”, “NaN” (Not a Number), “not available”, “-“, “unknown”, or other inconsistent entries in spreadsheets. For machine learning, these values cannot simply remain unmanaged in the dataset. A NaN value is not a medical measurement; it is an unresolved data state. Before modeling, each missing value must be either explained, coded according to a predefined rule, recovered from a valid source, excluded according to a documented criterion, or handled through an explicit missing-data strategy.

The most important point for clinicians is that an Excel file with many NaN values is not automatically usable for AI. If half of the patients have one group of laboratory tests and the other half have another group of tests, the dataset does not contain a stable predictor structure. For example, a dataset in which some patients have CRP, ESR, fibrinogen, and leukocyte counts, while others have only urea, creatinine, glucose, or liver enzymes, cannot be treated as a coherent laboratory dataset for one ML task unless this structure was intentionally designed and documented. The model requires comparable information across patients. A large spreadsheet with many empty cells is usually weaker than a smaller dataset with fewer variables collected consistently.

NaN values are especially problematic when they affect mandatory variables. A mandatory variable is one that is required for the main AI task. If the outcome, key predictors, clinical time point, or patient identifier is missing, the record may be unusable for the main analysis. If a mandatory laboratory marker is missing in a large proportion of eligible patients, the dataset may not be ready for the intended model. In that situation, the clinical team should not simply leave NaN values in the table. It should revise the data acquisition protocol, recover missing values from source systems, restrict the cohort, redefine the minimum common dataset, or move the variable from mandatory to optional.

Clinicians should also understand that not all NaN values have the same meaning. A missing test may mean that the test was not performed, was not clinically indicated, was unavailable in the hospital, was refused by the patient, was performed in another institution, was not extracted from the source system, or does not apply to that patient. These situations are clinically different. A missing CRP because the test was not clinically indicated is not the same as a missing CRP caused by data extraction failure. A missing follow-up score because the patient died before follow-up is not the same as a missing follow-up score because the patient was lost to follow-up. Therefore, NaN should be replaced by explicit missing-data codes whenever possible.

A practical missing-data coding system should be defined before data entry begins. For example, the dataset may use codes such as not_measured, not_clinically_indicated, unavailable, refused, not_applicable, lost_to_followup, and extraction_error. These codes should not be mixed with numerical values. They should be documented in the data dictionary and used consistently by all data collectors. This prevents the same situation from being recorded as an empty cell, “NA”, “NaN”, “unknown”, “not done”, “0”, or a free-text note by different people.

The use of “0” as a replacement for NaN should be avoided unless zero is truly the measured value. In medical datasets, zero may be clinically meaningful. It may indicate no pain, no lesions, no events, no dose, no comorbidities, or an actual measured value depending on the variable. If zero is used to mean missing, the dataset becomes ambiguous and may mislead the model. Missingness should be represented through explicit missing-data codes, not through values that may also have clinical meaning.

Before modeling, the technical team must decide how NaN values will be handled. Common options include complete-case analysis, variable exclusion, patient exclusion, imputation, missingness indicators, model-native missing-value handling, or subgroup analysis. However, these are methodological decisions that should be made only after the clinical meaning of missingness is understood. Recent work on prediction models using electronic health record data emphasizes that missingness is common and that missing-data strategies must be compatible with both model development and real-world prediction workflows [33,34]. Therefore, the clinical team should not leave NaN values unexplained and expect preprocessing to solve the problem automatically.

For predictor variables, NaN values may sometimes be handled through imputation or model-specific missing-value strategies, but this does not remove the need for documentation. If a laboratory value is missing because it was ordered only in severe patients, imputation may hide an important clinical pattern. If a test is missing because it was unavailable in one hospital, the missingness may reflect a site effect. If a variable is missing because it was not extracted, the problem is technical and should be corrected if possible. The reason for missingness determines whether the variable can be used safely in the main model.

For outcome variables, NaN values are more serious. A supervised AI model cannot learn a reliable target if the label is missing, vague, or unknown. A missing outcome should never be automatically treated as a negative outcome. For example, if 30-day readmission status is unknown, the patient should not be coded as “no readmission”. The correct code may be followup_incomplete, lost_to_followup, transferred, or outcome_unknown, depending on the situation. If too many outcomes are unknown, the dataset may be unsuitable for supervised modeling.

For follow-up variables, NaN values must be linked to the follow-up process. A missing follow-up result may mean that the patient missed the visit, was followed in another institution, died before the planned assessment, refused reassessment, or was not yet due for follow-up. These situations should be distinguished whenever possible. In longitudinal or prognostic studies, the dataset should record the planned follow-up time, the observed follow-up duration, the event status, and the reason why follow-up is incomplete.

The clinical team should perform a NaN audit before the dataset is sent for modeling. This audit should identify the percentage of missing values for each variable, the percentage of patients with missing mandatory variables, the variables missing by design, the variables missing only in specific subgroups, and the variables with unexplained missingness. Variables with high unexplained missingness should not be used as central predictors. Patients with missing mandatory variables should be reviewed according to predefined inclusion and exclusion rules. The audit should be documented and preserved as part of the dataset preparation process.

A practical NaN audit should answer the following questions for each important variable: Is this variable mandatory, recommended, or optional? How many values are missing? Why are they missing? Can the missing values be recovered from a valid source? Are they missing in a clinically meaningful subgroup? Should the variable remain in the main model, be moved to exploratory analysis, or be excluded? How will the remaining missing values be handled before modeling?

In practical terms, NaN values should never be treated as harmless empty cells. They are signals that the dataset is incomplete, inconsistently collected, or insufficiently documented. A dataset becomes AI-ready only when missing values are made explicit, clinically interpreted, and handled through a predefined strategy. Physicians and clinical researchers are best positioned to explain why values are missing; data scientists can then decide how those values should be treated computationally.

### 3.10. Prepare and Maintain a Data Dictionary from the Beginning

A medical dataset should not be collected as a spreadsheet whose meaning is known only to the person who created it. For an AI-ready dataset, every variable must be defined in a data dictionary before or during data collection. The data dictionary is the document that explains what each column, file, label, code, time point, and data source means. Without it, the dataset may be technically readable but clinically ambiguous.

The data dictionary should be prepared from the beginning of the study, not after the dataset has already been completed. If variable definitions are added retrospectively, the clinical team may discover too late that different people used the same column differently, that units were mixed, that time points were unclear, or that missing values were recorded inconsistently. A data dictionary prevents this by establishing common rules for data entry, interpretation, and later analysis.

For each variable, the data dictionary should include the variable name, clinical definition, data type, unit of measurement, allowed values, coding rule, source document or system, clinical time point, mandatory/recommended/optional status, missing-data rule, and role in the study. The role of the variable should state whether it is intended as a predictor, outcome, identifier, grouping variable, adjustment variable, metadata field, quality-control variable, or exploratory variable. This distinction is important because the same clinical information may have different functions depending on the AI task.

Variable names should be clear and stable. Names such as “CRP”, “score”, “status”, “image”, “result”, or “complication” are often insufficient because they do not specify timing, clinical meaning, or role. More informative names include CRP_at_admission, creatinine_preoperative, WBC_pre_treatment, CT_baseline, MRI_followup, outcome_30days, readmission_90days, or infection_confirmed_by_culture. These names help clinicians and data scientists understand what the variable represents without relying on informal explanations.

The data dictionary should define units and coding conventions explicitly. Laboratory values should include their measurement units. Categorical variables should include all allowed categories and their meaning. Binary variables should define what 0 and 1 represent. Ordinal variables should define the order and clinical interpretation of each level. Date variables should use a consistent format. Missing-data codes should be listed and explained. If a variable has been converted, transformed, harmonized, or derived from another variable, the transformation rule should be documented.

A data dictionary is also necessary for ethical and unbiased AI development. Recent work on clinical variables for AI-driven tools in vascular disease research emphasizes that structured data dictionaries are needed to ensure high-quality, unbiased, and ethically sound data input for AI models [35]. This is directly relevant to physician-led datasets, because bias and inconsistency can enter the dataset before any model is trained, simply through unclear variable definitions, inconsistent data extraction, or undocumented inclusion rules.

The data dictionary should also include metadata for non-tabular data. For imaging data, it should record modality, acquisition date, anatomical region, protocol, device or scanner information, file format, time point, label source, and linkage to patient and outcome. For physiological signals, it should record signal type, device, sampling frequency, duration, channels, acquisition conditions, segmentation rule, quality flags, and clinical time point. For textual data, it should record document type, source, date, de-identification status, intended use, and linkage to patient and outcome. These metadata are required because non-tabular files are not self-explanatory.

The data dictionary should support interoperability and reuse. A dataset may initially be used by one clinical team and one technical team, but later it may be extended, validated, shared, reused, or compared with other datasets. Standardized metadata make this possible. Recent work on the NFDI4Health Metadata Schema showed that structured metadata can improve the FAIRness of clinical, epidemiological, and public health research resources and support interoperability across services and adjacent domains [36]. Although a small clinical AI project does not need the same infrastructure as a national metadata repository, it should follow the same principle: data must be documented so that they can be understood and reused beyond the original collector.

The data dictionary should be maintained as a living document. When a new variable is added, a unit is changed, a code is corrected, or a missing-data category is introduced, the dictionary should be updated at the same time as the dataset. If multiple versions of the dataset exist, the corresponding version of the data dictionary should be preserved. This avoids situations in which the spreadsheet changes but the documentation no longer matches the data.

The clinical team should also use the data dictionary as a quality-control tool. During pilot data entry, the dictionary can reveal whether variables are unclear, whether allowed values are incomplete, whether data collectors interpret categories differently, or whether mandatory variables are unrealistic. If two clinicians cannot fill the same variable in the same way using the data dictionary, the definition is not yet precise enough for AI-ready collection.

A practical data dictionary entry should answer the following questions: What is the variable called? What does it mean clinically? What type of data is it? What unit or coding system is used? What values are allowed? Where does the information come from? When is it collected? Is it mandatory, recommended, or optional? How are missing values recorded? Is it a predictor, outcome, identifier, metadata field, or exploratory variable? Has it been transformed or derived from another field?

In practical terms, the data dictionary is the translation layer between medical reasoning and technical analysis. It allows clinicians to define the meaning of the data and allows data scientists to use the dataset without guessing. A dataset without a data dictionary may still be readable as a file, but it is not sufficiently documented for responsible AI development.

### 3.11. Perform a Pre-Modeling AI-Readiness Check Before Data Transfer

Before a medical dataset is transferred to a technical team or used for AI, ML, or DL model development, the clinical team should perform a pre-modeling AI-readiness check. This check is not a statistical validation of the future model. It is a clinical and data-preparation review intended to determine whether the dataset is sufficiently coherent, complete, documented, and temporally appropriate to justify model development.

The first element of this check is task clarity. The clinical team should confirm that the intended AI task is explicitly defined. It should specify the target, population, clinical decision point, and intended use clearly enough to guide dataset construction.

The second element is cohort consistency. The clinical team should verify that inclusion criteria, exclusion criteria, care setting, study period, and unit of analysis are documented. The unit of analysis should be applied consistently across all records, with explicit rules for repeated or multiple observations from the same patient.

The third element is minimum common dataset completeness. The team should verify that the mandatory variables defined in the acquisition protocol are available for eligible cases. Variables with substantial unexplained missingness should be reconsidered before data transfer, particularly when they are required for the main AI task.

The fourth element is temporal consistency. Each predictor should be checked against the intended prediction or classification moment. Variables recorded after the decision point, after diagnosis, after treatment, after discharge, or after outcome occurrence should not be used as predictors for earlier decisions. Rules for handling repeated measurements should also be predefined rather than selected retrospectively during modeling.

The fifth element is outcome reliability. The target outcome should have a clear clinical definition, a documented confirmation source, a measurement time point, and a label type. If the outcome is missing, vague, inconsistently assigned, or disconnected from the correct patient episode, the dataset is not ready for supervised learning. Outcome uncertainty should be documented rather than silently converted into a negative label.

The sixth element is documentation completeness. The dataset should include a data dictionary that defines each variable, its clinical meaning, data type, unit, allowed values, source, collection time point, missing-data rule, and role in the study. For non-tabular data, the documentation should also preserve the metadata and patient-level linkage required for interpretation.

The seventh element is missing-data resolution. The clinical team should perform a NaN audit before data transfer. The audit should distinguish missing predictors from missing outcomes, identify unexplained missingness in mandatory variables, and document whether missing information can be recovered from valid sources or must remain as a dataset limitation.

The eighth element is validation feasibility. Before modeling, the clinical team and technical team should verify whether the dataset can support meaningful evaluation. This includes the number of independent cases and outcome events, class imbalance, repeated records from the same patient, patient-level splitting where required, and the feasibility of internal or external validation. Recent guidance on predictive AI model evaluation emphasizes that medical AI models should be assessed across clinically meaningful domains, including discrimination, calibration, and clinical utility, rather than relying on isolated performance measures [37]. AI-readiness therefore includes not only whether a model can technically be trained, but also whether its performance can later be evaluated in a methodologically meaningful way.

The ninth element is deployment relevance. Even if the dataset is technically usable, the clinical team should ask whether it represents the setting in which the model would later be used. Datasets restricted to a selected subgroup, device, operator, hospital unit, or time period may still support exploratory modeling, but these characteristics should be documented as potential limitations to generalizability. Systematic evidence on clinical prediction model implementation and updating shows that validation, monitoring, and adaptation to real-world use are central steps after model development [38]. For this reason, the dataset should be reviewed for likely limitations before modeling begins.

The outcome of this pre-modeling review should be documented before the technical modeling phase begins. The checklist, scoring approach, and critical blocking criteria described below provide the structured mechanism for translating these findings into a final readiness decision.

### 3.12. Consider Dataset Size, Target Definition, Class Balance, and Model Type Before Modeling

Dataset size is a critical component of AI-readiness, but it cannot be evaluated without a clearly defined target. Before asking whether a dataset is large enough for ML or DL, the clinical team must define what the model is expected to predict or classify, what the positive and negative classes are, when the target is measured, and how the target is confirmed. A dataset with 1000 patients may be insufficient if the target event occurs in only 20 patients, while a smaller dataset may be useful for exploratory ML if the target is frequent, clearly defined, and reasonably balanced across classes.

The target should therefore be written in operational clinical terms. Vague targets such as “poor evolution”, “severe patient”, “complicated case”, or “treatment failure” are not sufficient for dataset-size planning. More useful targets include “30-day postoperative infection confirmed by clinical diagnosis, microbiology, or surgical revision”, “ICU admission within 7 days after hospital admission”, “readmission within 30 days after discharge”, or “treatment response at 6 months defined as at least 50% reduction in a validated symptom score”. Once the target is defined, the clinical team can count how many independent cases exist in each target class.

For AI, ML, and DL studies, the relevant sample size is not only the total number of records. The most important quantities are the number of independent patients or clinical cases, the number of target events, the number of cases in each class, the number of predictors or input features, and the amount of data that can be separated for training, validation, and testing. A dataset may appear large but still be weak if most records come from a small number of patients, if one class dominates the dataset, or if the number of outcome events is too low for the intended model.

There is no universal minimum number of patients that guarantees a good AI model. Required sample size depends on the clinical task, target frequency, number of predictors, model complexity, class distribution, missing data, label reliability, and validation strategy. Recent methodological guidance emphasizes that inadequate sample size can lead to unstable models, imprecise performance estimates, poor calibration, lower clinical utility, and potentially misleading clinical conclusions [39,40]. For this reason, physicians should not ask only whether a dataset is “large enough” in general, but whether it is large enough for the specific target, model type, and validation plan.

Table 3 provides practical planning ranges for clinical researchers. These ranges are not statistical guarantees and should not replace formal sample-size calculation. They are intended as pragmatic warnings for physicians who are planning data collection and need to understand the difference between descriptive, exploratory, and more credible AI-oriented datasets.

For tabular ML studies using clinical variables, laboratory markers, comorbidities, treatment variables, and outcomes, the number of independent patients and the number of target events are central. A dataset with 1000 patients may still be weak if only 20 patients have the outcome of interest. Similarly, a dataset with 200 patients may be useful for exploratory analysis if the target is balanced, the predictor set is limited, clinically justified, and proportionate to the number of usable cases and target events, the variables are consistently collected, and the aim is clearly described as pilot or feasibility work. The total number of patients is therefore less informative than the number of usable records per target class.

For CNN and other DL models trained on images, the data requirement is usually higher than for simple tabular ML models. CNNs can learn complex visual patterns directly from images, but they can also overfit small, homogeneous, or poorly labeled datasets. The relevant number is not only the number of images, but the number of independent patients, lesions, examinations, and labeled cases per class. A folder with 5000 images from 200 patients should not be interpreted as 5000 independent clinical cases. If multiple images from the same patient are split across training and testing sets, the model may be evaluated on information from patients it has effectively already seen.

In imaging studies, the required dataset size also depends on whether the model is trained from scratch, fine-tuned from a pretrained model, or used as a feature extractor. Training a CNN from scratch usually requires substantially more labeled data than transfer learning. However, transfer learning does not remove the need for independent cases, balanced labels, reliable annotations, patient-level splitting, and clinically meaningful validation. Biomedical imaging research continues to emphasize data scarcity and the limited availability of large, well-annotated datasets as major constraints for DL generalization [41]. Therefore, small imaging datasets should usually be framed as pilot, feasibility, or transfer-learning studies rather than as definitive evidence for clinical deployment.

Class balance must be evaluated together with the target. A binary classification dataset with 2000 positive cases and 20 negative cases is not a strong binary classification dataset simply because it contains 2020 records. It is a highly imbalanced dataset. The limiting factor is the minority class. In such a dataset, a model may appear accurate by mostly predicting the majority class, while performing poorly on the clinically important minority class. Sensitivity, specificity, precision, recall, F1 score, calibration, and class-specific performance should therefore be interpreted in relation to the number of cases in each class, not only in relation to the total sample size.

For binary classification, the clinical team should count both positive and negative cases before modeling. For multiclass classification, the team should count the number of independent cases in every class. For ordinal severity models, the team should check whether all severity levels are represented. For prognostic models, the team should count both event and non-event cases within the defined follow-up window. If the minority class is very small, the dataset may not support reliable classification for that class. In such situations, the study should be redesigned, more cases should be collected, categories should be clinically reconsidered, or the analysis should be downgraded to exploratory status.

Imbalance correction methods should not be treated as a substitute for adequate data collection. Oversampling, undersampling, class weighting, synthetic data generation, and other imbalance-handling methods may be useful in some contexts, but they do not create new independent clinical information. Recent simulation evidence in clinical prediction modeling shows that class imbalance corrections may harm calibration and can lead to risk overestimation when reliable individual risk estimates are needed [42]. Therefore, the first response to severe imbalance should be clinical and methodological review of the dataset, not automatic computational correction.

The number of predictors should also be considered. A dataset with a small number of patients cannot safely support a large number of candidate predictors. If the clinical team collects 50 variables for 120 patients, the risk of overfitting is high, especially if the target event is rare. For tabular ML, physicians should prefer a smaller number of clinically justified predictors collected consistently over a large number of irregular variables. For imaging DL, physicians should remember that the model may extract thousands of internal features from images, making independent sample size, annotation quality, and validation design even more important.

Validation planning must be included in dataset-size assessment. Data are needed not only for training, but also for internal validation, tuning, testing, and ideally external validation. If the dataset is already small, splitting it into training, validation, and test sets may leave too few cases in each subset, especially in the minority class. For imaging datasets, splitting must be performed at the patient level, not only at the image level. For longitudinal datasets, splitting must respect patient identity and time. If a dataset cannot support a defensible validation strategy, it should not be presented as AI-ready for model development.

A practical dataset-size and class-balance review should answer the following questions before modeling: What is the target? How is the target confirmed? When is the target measured? Which class is positive and which class is negative? How many independent patients are included? How many records come from the same patient? How many positive and negative cases are available? How many cases exist in each class or severity level? How many predictors are planned? How many mandatory variables contain NaN values? Is the minority class large enough for meaningful evaluation? Can the dataset be split without leaking patient information between training and testing? Is the dataset suitable for tabular ML, CNN/DL, or only exploratory analysis?

In practical terms, dataset size and class balance determine whether an AI project is realistic. A clean dataset with 300 well-defined patients may be more valuable for a simple exploratory ML task than a large spreadsheet with thousands of inconsistent records, unclear targets, unexplained NaN values, and extreme class imbalance. Conversely, CNN or DL studies on images usually require larger, well-labeled, patient-level independent datasets, especially when the goal is to train or fine-tune visual models. Physicians should therefore plan the target, dataset size, class distribution, and validation strategy before modeling, not after poor performance or unstable results appear.

## 4. AI-Readiness Checklist and Scoring Approach

After the practical data-collection requirements have been defined, clinical teams need a structured way to verify whether a dataset is sufficiently prepared for AI, ML, or DL model development. This section converts the Clinical AI-Readiness Guide into two practical tools: a checklist that helps physicians identify whether the essential preparation requirements are met, and a scoring approach that summarizes the maturity of the dataset before modeling begins. These tools are not intended to certify model validity or replace statistical, clinical, or methodological review. Their purpose is to make dataset weaknesses visible early, before time and resources are invested in modeling.

### 4.1. Purpose of the AI-Readiness Checklist

The AI-readiness checklist is designed as a pre-modeling verification tool for clinical teams preparing medical datasets for AI-based studies. Its purpose is not to determine whether a future model will be accurate, clinically useful, or ready for implementation. Instead, it verifies whether the dataset has the minimum structure, documentation, and clinical coherence required before model development can reasonably begin.

The checklist should be completed after the data acquisition protocol has been defined and before the dataset is transferred to the technical team. It can also be used during pilot data entry, retrospective data extraction, or early dataset cleaning. At each stage, the checklist helps the clinical team identify whether essential elements are missing, unclear, inconsistently collected, or insufficiently documented.

The checklist focuses on practical dataset-readiness questions. It asks whether the clinical task is explicit, whether the cohort is defined, whether the minimum common dataset exists, whether mandatory variables are sufficiently complete, whether the outcome is clearly specified, whether measurements are collected at consistent time points, whether NaN values are explained, whether units and coding are standardized, whether non-tabular data are linked and documented, whether a data dictionary exists, and whether the dataset can support a meaningful validation plan.

This checklist is especially useful because many dataset weaknesses are not visible from the number of patients or columns alone. A dataset may contain many variables but still be weak if each patient has a different subset of measurements. It may contain many images but still be unusable if they are not linked to patients, time points, and labels. It may contain follow-up information but still be unsuitable for prognosis if the follow-up window is inconsistent or undocumented. The checklist is intended to reveal these weaknesses before modeling begins.

The checklist should be interpreted clinically, not mechanically. A dataset does not become AI-ready simply because many checklist items are marked as complete. Some requirements are essential for the main task. For example, an undefined outcome, an unclear unit of analysis, unexplained NaN values in mandatory predictors, mixed measurement time points, or absent patient-level linkage may block responsible model development even if other parts of the dataset are well prepared.

In practical terms, the checklist acts as a communication tool between clinicians and data scientists. Instead of sending only a spreadsheet, image folder, or exported database, the clinical team can send the dataset together with the completed checklist and data dictionary. This allows the technical team to understand what the dataset contains, which variables are reliable, which variables are exploratory, where the main risks are, and whether modeling should proceed, be delayed, or remain exploratory.

### 4.2. Checklist Domains

The AI-readiness checklist is organized into ten domains. Each domain corresponds to a core preparation requirement that should be reviewed before model development begins. The domains follow the same logic as the data-collection guide: the clinical task must be clear, the cohort must be defined, the same core variables must be collected consistently, the outcome must be reliable, variables must be measured at meaningful time points, NaN values must be explained, data must be standardized, non-tabular files must be linked and documented, a data dictionary must be available, and the dataset must support a defensible validation plan.

Table 4 presents the proposed checklist domains. Each domain can be marked as complete, partially complete, or incomplete. A complete item means that the requirement is clearly defined and documented. A partially complete item means that the requirement exists but has limitations that should be corrected or explicitly reported. An incomplete item means that the dataset is not yet prepared in that domain. A fillable spreadsheet version of the checklist is provided as Table S1 in the Supplementary Materials.

The checklist should be used as a structured review rather than as a purely administrative form. A dataset may be strong in one domain and weak in another. For example, laboratory data may be well standardized, but the outcome may be vague. Imaging files may be numerous, but patient-level linkage may be missing. A dataset may contain a large number of patients, but mandatory variables may be absent in many records. The purpose of the checklist is to identify these weaknesses before modeling begins.

The checklist is also useful during pilot data entry. Before collecting the full dataset, the clinical team can complete the checklist on a small sample of records. This pilot review can reveal that some mandatory variables are not consistently available, that the outcome definition is difficult to apply, that units are mixed, or that time points are unclear. Correcting these problems early is easier than repairing the dataset after hundreds or thousands of records have already been collected.

In practice, the checklist should be completed by the clinical team and reviewed together with the technical team. The clinical team is responsible for defining the medical meaning of the data, while the technical team can identify modeling risks, leakage risks, validation constraints, and preprocessing requirements. This shared review helps determine whether the dataset can proceed to model development, requires revision, or should remain exploratory.

### 4.3. AI-Readiness Scoring Approach

To make the checklist easier to apply in clinical practice, each checklist domain can be scored using a simple three-level scale. The purpose of the score is not to produce a statistical measure of model quality, but to summarize the maturity of the dataset before model development begins. The score helps clinical teams identify whether the dataset is ready for modeling, requires revision, or should remain exploratory.

Each of the ten checklist domains is scored as follows:

0 = incomplete;

1 = partially complete;

2 = complete.

The three-level 0–2 scale was selected as a deliberately simple ordinal representation of dataset readiness: 0 indicates that the requirement is absent or insufficiently defined, 1 indicates that it is present but incomplete, and 2 indicates that it is sufficiently defined and documented for pre-modeling assessment. The purpose of this scale is practical interpretability rather than measurement precision. All ten domains receive equal numerical weighting to preserve transparency and ease of use and because empirical evidence supporting differential domain weights is not yet available. Equal weighting should not be interpreted as implying equal methodological importance. More consequential deficiencies are addressed separately through the critical blocking criteria, so a high total score cannot compensate for a fundamental problem such as an undefined outcome, temporal leakage, or absent patient-level linkage.

The total AI-readiness score therefore ranges from 0 to 20 points. A higher score indicates that the dataset is better prepared for AI, ML, or DL analysis. However, the score should not be interpreted mechanically. It is a structured screening tool, not a substitute for clinical judgment, statistical review, or methodological assessment.

A domain should receive 2 points when the requirement is clearly defined, consistently applied, and documented. For example, the outcome domain receives 2 points when the outcome has explicit clinical criteria, a reliable confirmation source, a defined measurement time point, and a clear label type. The missing-data domain receives 2 points when NaN values are coded, explained, and documented according to predefined rules.

A domain should receive 1 point when the requirement is present but incomplete. For example, the cohort may be generally defined, but the unit of analysis may remain unclear. Laboratory variables may have units, but not all time points may be documented. Imaging files may be linked to patient identifiers, but acquisition metadata or label sources may be incomplete. A score of 1 means that the dataset may still be usable, but the limitation must be corrected or reported before modeling.

A domain should receive 0 points when the requirement is absent or too unclear to support responsible model development. For example, a dataset receives 0 points for outcome definition if the outcome is vague, subjective, missing, or inconsistently assigned. It receives 0 points for missing-data handling if NaN values are left unexplained. It receives 0 points for non-tabular data if imaging, signal, text, or pathology files are stored without reliable linkage to patient identifiers, time points, labels, or outcomes.

The score should be calculated after the checklist has been completed. The clinical team should first review each domain, assign a score of 0, 1, or 2, and document the reason for the score. The scoring notes are important because two datasets may have the same total score but very different weaknesses. For example, one dataset may lose points because optional imaging metadata are incomplete, while another may lose points because the outcome is weakly defined. These two situations do not have the same methodological importance.

The proposed score ranges are pragmatic, expert-informed categories rather than empirically derived cut-off values. They were selected to represent progressively higher levels of completion across the ten checklist domains. Scores of 0–7 indicate that substantial elements of dataset preparation remain incomplete; scores of 8–12 indicate sufficient structure for exploratory use but persistent limitations; scores of 13–16 indicate that most core requirements are substantially addressed, although important limitations remain; and scores of 17–20 require a high level of completion across the checklist before the dataset can be considered ready for model development, provided that no critical blocking criterion is present. These thresholds were not derived from empirical calibration, outcome-based optimization, or validation against an external reference standard and should therefore not be interpreted as validated cut-off values.

The proposed interpretation of the total score is

0–7 points: not ready for AI modeling;

8–12 points: exploratory only;

13–16 points: ML-ready with important limitations;

17–20 points: AI-ready for model development, pending clinical, statistical, and methodological review.

A dataset classified as “not ready” lacks essential elements required for AI-based analysis. Such a dataset should not be sent directly for model development. The appropriate next step is to clarify the clinical question, define the cohort and outcome, construct a minimum common dataset, resolve major NaN problems, and prepare a data dictionary.

A dataset classified as “exploratory only” may contain useful clinical information, but it has substantial limitations that prevent reliable model development. It may support descriptive analysis, feasibility assessment, pilot exploration, or planning of a future prospective dataset. However, it should not be used to make strong claims about model performance or clinical utility.

A dataset classified as “ML-ready with important limitations” has a usable structure but still contains constraints that must be documented. These may include moderate missingness, limited sample size, class imbalance, single-center origin, incomplete metadata, partial follow-up, or limited validation options. Such a dataset may support model development, but the limitations should shape the analysis plan and the interpretation of results.

A dataset classified as “AI-ready for model development” has a defined task, coherent cohort, reliable outcome, consistent mandatory predictors, documented timing, explained missing values, standardized variables, linked non-tabular data where relevant, a data dictionary, and a plausible validation plan. This classification does not guarantee that the future model will perform well. It only indicates that the dataset is sufficiently prepared to begin responsible model development.

The scoring approach should be used as a decision-support instrument. It helps clinical teams decide whether to proceed with modeling, revise the dataset, collect additional information, restrict the analysis, or keep the project exploratory. Its main value is not the numerical score itself, but the structured discussion it creates between clinicians and technical teams before modeling begins.

**Figure 4:**
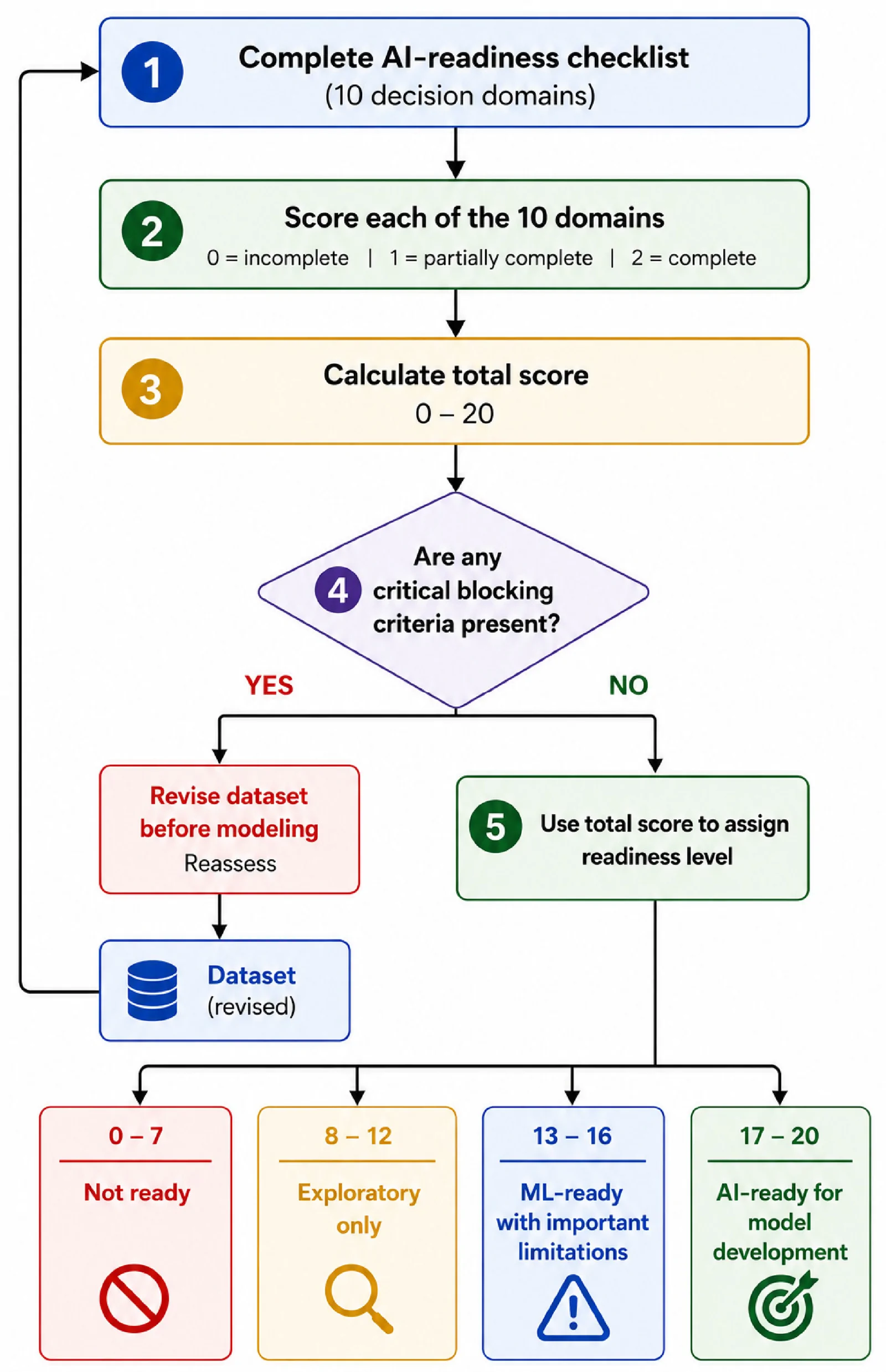
AI-readiness decision tree integrating checklist completion, domain scoring, blocking criteria, reassessment, and final readiness classification.

Figure 4 presents a decision-tree summary of how the checklist score should be combined with critical blocking criteria before assigning the final readiness level. The figure makes explicit that scoring and blocking criteria are complementary: the score places the dataset in a readiness band, while blocking criteria determine whether progression to modeling is methodologically acceptable.

The diagram should be read from top to bottom. After the checklist is completed, each of the ten domains is scored from 0 to 2, the total score is calculated, and the dataset is checked for critical blocking criteria. If such a criterion is present, the dataset should be revised before modeling, regardless of its numerical score, and the revised dataset should then be reassessed by repeating the checklist and scoring process. If no blocking criterion is present, the readiness level can be assigned using the predefined score ranges. This logic prevents premature model development on datasets that appear numerically acceptable but still contain fundamental clinical, temporal, documentation, linkage, or validation flaws.

### 4.4. Critical Blocking Criteria

The AI-readiness score should always be interpreted together with critical blocking criteria. Some dataset weaknesses are severe enough to prevent responsible AI model development, even when the total checklist score appears acceptable. These criteria identify situations in which modeling should be delayed until the dataset is corrected, clarified, or restricted to an exploratory analysis.

The critical blocking criteria were selected using a literature-informed and expert-informed approach rather than through formal expert consensus or empirical validation. A dataset weakness was designated as a blocking criterion when it could fundamentally compromise the definition, clinical interpretation, temporal validity, traceability, or evaluability of the intended AI task in a way that could not be compensated for by higher scores or greater completeness in other checklist domains. Published evidence supports the methodological importance of several of these conditions, including reliable outcome and annotation procedures [16], prevention of temporal and label leakage [17,20], explicit interpretation and handling of missing data [8,33], structured dataset documentation and data dictionaries [19,35], and the feasibility of meaningful model evaluation and validation [37,39]. The resulting criteria therefore integrate published methodological evidence with the multidisciplinary clinical and technical expertise and practical experience represented within the author team. They are intended as conservative pre-modeling safeguards rather than as empirically validated exclusion rules.

The first blocking criterion is an undefined clinical task. If the dataset does not specify what the future model is intended to predict, classify, detect, monitor, or support, model development should not begin. Without a defined task, it is impossible to determine which patients should be included, which variables are relevant, which time points are appropriate, or which outcome should be used.

The second blocking criterion is an unclear unit of analysis. If the dataset mixes patients, admissions, visits, images, lesions, procedures, or signal segments without clear rules, the records cannot be interpreted correctly. This is especially important when multiple records come from the same patient. A dataset with many images or visits may appear large, but it may still represent a small number of independent patients.

The third blocking criterion is an unreliable or undefined outcome. Supervised AI models require a target label. If the outcome is vague, inconsistently assigned, missing for many records, or not linked to a confirmation source, the dataset should not be used for supervised modeling. Labels such as “severe”, “complicated”, “improved”, or “poor outcome” are insufficient unless they are linked to explicit clinical criteria.

The fourth blocking criterion is excessive missingness in mandatory variables. If key predictors required for the main AI task are missing in a large proportion of eligible patients, the dataset is not ready for that task. This is especially problematic when NaN values are unexplained. A dataset should not proceed to modeling if the technical team cannot distinguish between values that were not measured, not clinically indicated, unavailable, not applicable, lost to follow-up, or not extracted.

The fifth blocking criterion is mixed measurement timing. If the same column contains values collected at different clinical time points, the variable is not coherent. For example, CRP measured at admission, after treatment, and at discharge should not be merged into a single CRP column. Similarly, baseline imaging and follow-up imaging should not be treated as equivalent unless the study is explicitly designed to analyze repeated time points.

The sixth blocking criterion is data leakage risk. If predictors include information that would not be available at the intended prediction moment, modeling should be delayed until the predictor set is corrected. Examples include using discharge diagnoses for admission-time prediction, post-treatment markers for baseline treatment-response prediction, or follow-up information as a baseline predictor.

The seventh blocking criterion is absent linkage between non-tabular files and clinical records. Imaging, signal, text, pathology, or other external files must be linked to the correct patient, time point, label, and outcome. A folder of images or signals without reliable patient-level linkage is not sufficient for AI model development.

The eighth blocking criterion is absence of a data dictionary. If variable meanings, units, coding rules, time points, missing-data codes, and variable roles are not documented, the dataset is not sufficiently interpretable. In such cases, the technical team may preprocess or model variables without understanding their clinical meaning.

The ninth blocking criterion is lack of validation feasibility. If the dataset is too small, extremely imbalanced, heavily duplicated, or structured in a way that prevents patient-level splitting, meaningful model evaluation may not be possible. In such cases, the dataset may still be useful for exploratory analysis, but not for strong claims about model performance.

When any critical blocking criterion is present, the dataset should not be classified as AI-ready for model development, regardless of the total score. The clinical team should first correct the dataset, revise the research question, restrict the cohort, redefine the outcome, recover missing information, separate time points, or downgrade the study to exploratory status.

In practical terms, the blocking criteria protect the study from premature modeling. They prevent a situation in which a dataset receives a moderate numerical score but still contains a fundamental flaw that invalidates the AI task. The score summarizes readiness, but the blocking criteria determine whether modeling can responsibly begin.

### 4.5. Documenting the Readiness Decision

The final step in the checklist process is to document the readiness decision before model development begins. The result of the checklist and scoring process should not remain an informal discussion between clinicians and data scientists. It should be recorded as part of the dataset documentation, together with the data acquisition protocol, data dictionary, missing-data audit, and any notes on limitations.

The readiness decision should state whether the dataset is not ready, exploratory only, ML-ready with important limitations, or AI-ready for model development. This decision should be accompanied by a short explanation. For example, a dataset may be classified as exploratory because the outcome is available but follow-up is incomplete, or as ML-ready with limitations because the minimum common dataset is coherent but the cohort is single-center and moderately imbalanced. The explanation is important because the same numerical score may hide different types of weaknesses.

The clinical team should also document which limitations were accepted and which were corrected before modeling. Some limitations can be resolved by recovering missing laboratory values, separating mixed time points, clarifying outcome definitions, completing metadata, or revising the data dictionary. Other limitations cannot be fully corrected, such as small sample size, retrospective design, single-center origin, or unavailable follow-up. These limitations should not be hidden; they should be explicitly recorded and later reflected in the analysis plan and manuscript interpretation.

A readiness decision should include at least five elements: the total AI-readiness score, the readiness level, the presence or absence of blocking criteria, the main dataset limitations, and the action recommended before modeling. The recommended action may be to proceed to model development, revise the dataset, collect additional data, restrict the cohort, move some variables to exploratory analysis, or stop the AI component and use the dataset only for descriptive clinical analysis.

This documentation also improves communication between clinical and technical teams. When the technical team receives a dataset together with a checklist, score, data dictionary, and readiness decision, it can understand which variables are reliable, which variables are exploratory, where missingness remains problematic, which outcomes are usable, and which assumptions must not be violated during modeling. This reduces the risk that technical preprocessing decisions will be made without clinical context.

The readiness decision should be updated if the dataset changes. If additional patients are added, missing values are recovered, variables are recoded, labels are corrected, or follow-up is completed, the checklist and score should be recalculated. This creates a transparent audit trail from the initial dataset to the final modeling dataset. In collaborative medical AI projects, such versioned documentation is especially useful because several clinicians and technical contributors may work on the dataset at different stages.

In practical terms, the checklist and scoring approach transform AI-readiness from a vague impression into a documented decision. Instead of asking whether the dataset “looks good enough”, the clinical team can state what is complete, what is incomplete, what remains risky, and what action is justified. This makes the transition from clinical data collection to technical model development more transparent, reproducible, and clinically responsible.

### 4.6. Illustrative Worked Example of the Clinical AI-Readiness Assessment

To illustrate how the proposed Clinical AI-Readiness Guide can be applied in practice, consider a hypothetical retrospective dataset prepared for the development of a model predicting 30-day postoperative complications in patients undergoing orthopedic surgery. This example is entirely illustrative, contains no real patient data, and is intended to demonstrate the operational use of the checklist, scoring approach, critical blocking criteria, and final readiness classification. It should not be interpreted as an empirical validation of the proposed framework.

The hypothetical dataset contains 420 patients treated during a predefined study period, with each patient contributing one eligible surgical episode and one corresponding dataset record. The intended AI task is to predict, before surgery, whether a patient will develop a predefined postoperative complication within 30 days. The candidate predictor set includes demographic characteristics, relevant comorbidities, preoperative laboratory measurements, baseline imaging information, and other clinical variables intended to be available before the prediction moment. The outcome is defined using explicit clinical criteria and is determined from documented 30-day follow-up information.

The initial checklist assessment identifies several strengths. The clinical task, target population, prediction moment, study period, unit of analysis, and outcome definition are documented. Laboratory units, categorical coding, identifiers, and date formats have been standardized. The dataset also preserves patient-level independence and can support an internal validation strategy.

However, several limitations remain. Some mandatory laboratory variables are unavailable for a proportion of eligible patients, the reasons for several missing values have not yet been documented, imaging examinations are linked to the corresponding patients but have incomplete acquisition metadata, and the data dictionary does not yet provide complete definitions, timing information, and missing-data rules for all variables.

A more important problem is identified during the measurement-timing assessment. The dataset contains a variable labeled simply as CRP. Review of the hypothetical acquisition records shows that, for some patients, this value represents a preoperative measurement, whereas for others it represents an early postoperative measurement. Because the intended prediction is made before surgery, postoperative CRP values would not have been available at the prediction moment. Combining these measurements within the same predictor therefore violates the temporal eligibility rule and creates a potential data-leakage pathway.

Table 5 summarizes the initial assessment, the corrective actions indicated by the framework, and the subsequent reassessment after the hypothetical corrections have been applied.

The initial total score is 14/20, placing the dataset within the proposed “ML-ready with important limitations” range. The numerical score alone, however, does not determine whether model development should proceed. The presence of mixed preoperative and postoperative measurements constitutes a critical blocking criterion because information unavailable at the intended prediction moment has entered the candidate predictor set. Consequently, despite the numerical score of 14/20, the appropriate initial readiness decision is that model development should not proceed until the temporal inconsistency has been resolved.

The framework therefore directs the clinical team back to the relevant dataset-preparation steps rather than directly to model development. In this illustrative example, CRP measurements are separated according to their clinical time point, and only preoperative measurements remain eligible for the intended prediction task. Missing laboratory values are reviewed and assigned explicit missingness categories whenever their causes can be established from the available source information. Imaging metadata are completed where possible, and the data dictionary is updated to document variable definitions, timing, coding conventions, and missing-data rules. Information that is genuinely unavailable is not reconstructed or imputed at this stage solely to improve the readiness score; instead, the corresponding limitation remains explicitly documented.

Following these corrections, the checklist is completed again. The measurement-timing, missing-data, non-tabular-data, and data-dictionary domains improve, increasing the total score from 14/20 to 19/20. The minimum common dataset remains partially complete because some laboratory information cannot be recovered for all eligible patients. This limitation therefore remains visible in the reassessment rather than being artificially removed. However, the temporal blocking criterion has been resolved, and no other critical blocking criterion is present. Under the proposed scoring interpretation, the revised dataset would therefore be classified as “AI-ready for model development, pending clinical, statistical, and methodological review.”

This worked example illustrates several intended properties of the proposed framework. First, the readiness score and critical blocking criteria are complementary rather than interchangeable. A relatively high numerical score cannot compensate for a fundamental methodological problem such as temporal leakage, an unreliable outcome, absent patient-level linkage, or another critical blocking condition. Second, the checklist is intended not only to assign a readiness category but also to identify the specific dataset-preparation steps that require correction before modeling. Third, reassessment after correction provides a transparent way to document how the readiness decision changes as identified weaknesses are addressed.

The example demonstrates the operational logic and practical application of the Clinical AI-Readiness Guide but does not establish measurement validity, inter-rater reliability, optimal domain weighting, or empirically derived score thresholds. Formal evaluation of these properties across different clinical specialties, data types, institutional settings, and user groups remains necessary in future validation studies.

## 5. Discussion

This Technical Note proposes a physician-facing approach for preparing medical datasets before AI, ML, or DL model development. The central argument is that medical AI-readiness begins before algorithm selection. It begins when the clinical question, cohort, outcome, variables, measurement timing, missing-data logic, documentation rules, and validation context are defined. In this sense, dataset preparation is not a secondary technical step; it is a methodological condition for responsible medical AI research.

The proposed Clinical AI-Readiness Guide addresses a common gap in physician-led AI projects. Clinical teams often possess valuable routine data, but these data are not automatically suitable for AI modeling. Readiness depends not on data volume alone, but on whether clinical and non-tabular data are consistently structured, temporally defined, documented, and reliably linked to the relevant patient, clinical context, and outcome.

A key contribution of the proposed framework is the emphasis on uniform data acquisition. For many clinical AI studies, a smaller dataset with fewer variables collected consistently may be more useful than a large dataset with many irregularly completed columns. This is particularly relevant for laboratory-based datasets, where some biomarkers may be measured only in selected patients because of disease severity, clinical suspicion, or local diagnostic pathways. If these differences are not documented, the model may learn patterns of medical testing rather than patterns of disease. For this reason, the guide recommends defining a minimum common dataset before collection begins and separating mandatory, recommended, and optional variables.

Another important contribution is the explicit treatment of time. Because the clinical meaning and predictor eligibility of a variable depend on when it becomes available, mixing measurements from different stages of care can create clinically invalid predictors and data leakage. Recent evidence on clinical AI models has shown that leakage and inappropriate feature use can artificially improve apparent model performance and create misleading impressions of clinical utility [20].

The proposed checklist and scoring approach are intended to operationalize the guide. They do not certify model validity and do not replace clinical, statistical, or methodological review. Their role is to provide a structured pre-modeling assessment of whether the dataset is sufficiently prepared to justify model development.

The same intermediate step is relevant from a risk-management and traceability perspective. Recording unresolved dataset-level limitations before modeling creates a transparent account of the assumptions and residual risks that may later affect model validity, interpretability, accountability, and safe clinical use.

This preparation layer complements existing reporting guidelines rather than replacing them. Guidelines such as TRIPOD+AI, SPIRIT-AI/CONSORT-AI, CLAIM, and STARD-AI improve the reporting and appraisal of AI studies, prediction models, diagnostic accuracy studies, clinical trials, and imaging AI research [10,11,12,13]. The present guide focuses on an earlier practical stage: what physicians should collect, structure, label, and document before model development begins. In this sense, the framework can help clinical teams prepare datasets that are more likely to be reportable, interpretable, and methodologically defensible later.

The proposed guide also complements healthcare data-quality and data-standardization initiatives that operate at related but distinct levels. The FAIR principles establish broad requirements for making research data findable, accessible, interoperable, and reusable, but they do not define the clinical and task-specific decisions required to determine whether a dataset is suitable for a particular AI application [43]. The OMOP Common Data Model supports the harmonization of heterogeneous observational health data into a standardized structure and vocabulary, whereas the Clinical AI-Readiness Guide focuses on decisions that should be clarified before or during dataset preparation, including task definition, outcome specification, predictor eligibility, measurement timing, and missing-data logic [44]. Similarly, the OHDSI Data Quality Dashboard evaluates conformance, completeness, and plausibility in OMOP-standardized datasets, while the present guide addresses an earlier physician-facing stage in which the clinical meaning, temporal eligibility, documentation, and intended use of the data must first be established [45]. NIH initiatives on AI-ready clinical research data likewise emphasize structured data preparation and fitness for downstream AI/ML use [4]. The Clinical AI-Readiness Guide therefore does not replace or duplicate these approaches; rather, it provides a task-specific pre-modeling layer that can precede and complement subsequent standardization, data-quality assessment, interoperability, and reuse.

The guide is also relevant for collaboration between clinicians and data scientists. By documenting variable roles, measurement timing, missingness, outcome definitions, and data provenance before data transfer, the proposed acquisition protocol, data dictionary, NaN audit, checklist, and readiness decision help preserve clinical meaning during technical preprocessing.

The framework has practical implications for different types of medical data. Across tabular, laboratory, imaging, physiological-signal, and textual data, it requires the clinical and technical context needed to interpret each modality consistently, including appropriate timing, linkage, metadata, and documentation. This broad scope is necessary because medical AI is increasingly multimodal, and weaknesses in one modality can compromise the entire dataset.

A further implication concerns expectations about AI feasibility. The proposed scoring approach may help physicians decide whether an AI project is realistic at the current stage. Some datasets may be classified as not ready because the outcome is undefined, mandatory variables are missing, or patient-level linkage is absent. Others may be appropriate only for exploratory analysis or feasibility assessment. This classification can prevent premature claims about AI performance and can guide the design of future prospective data collection. It also encourages honest reporting of dataset limitations rather than treating preprocessing as a hidden technical solution.

The proposed framework has limitations. First, it is a practical guide rather than a validated measurement instrument. The checklist and score are intended to support structured decision-making, but they require further empirical evaluation across clinical specialties, data types, and institutional settings. Second, the scoring thresholds are pragmatic and should not be interpreted as universal cutoffs. Third, the guide does not solve all methodological problems in medical AI, such as model calibration, external validation, fairness, clinical utility, or deployment monitoring. These remain essential steps after dataset preparation and model development [37,38].

Another limitation is that the framework cannot eliminate constraints inherent to retrospective data. In many real-world settings, some variables were never collected, time points were not standardized, imaging protocols changed, or follow-up was incomplete. In such cases, the guide can help identify limitations and classify the dataset appropriately, but it cannot reconstruct information that was never recorded. This reinforces the importance of applying AI-readiness principles as early as possible, ideally during prospective study planning or before retrospective extraction begins.

Despite these limitations, the proposed guide offers a practical contribution for clinical researchers. By translating general AI-readiness principles into concrete pre-modeling decisions, it provides a structured way to identify and address dataset limitations before technical model development begins.

## 6. Conclusions

Medical AI studies should not begin with algorithm selection, but with the preparation of clinically meaningful and technically usable data. A dataset becomes AI-ready only when the clinical question is explicit, the cohort is defined, the outcome is reliable, the same core variables are collected consistently, measurement time points are documented, NaN values are explained, units and coding rules are standardized, non-tabular files are linked to patient-level information, and a data dictionary is available.

This Technical Note proposed a physician-facing Clinical AI-Readiness Guide for preparing medical datasets before AI, ML, or DL model development. The guide emphasizes practical decisions that clinical researchers can control during data acquisition, retrospective extraction, and early dataset organization. It also introduces a checklist and scoring approach that help clinical teams identify whether a dataset is not ready, exploratory only, ML-ready with limitations, or sufficiently prepared for model development.

The central message is that a large spreadsheet, image folder, signal archive, or collection of clinical notes is not automatically suitable for AI. For reliable medical AI research, data must be collected according to a predefined protocol, using a minimum common dataset, consistent clinical time points, explicit outcome definitions, documented missing-data rules, and clear linkage between patients, variables, files, and outcomes.

By making these requirements explicit, the proposed guide can improve collaboration between physicians and technical teams, reduce preventable dataset limitations, and support more responsible development of AI models in medical research. Future work should evaluate the checklist and scoring approach across clinical specialties, data types, and institutional settings.

## Figures and Tables

**Table 1:** Minimum elements of a medical data acquisition protocol for AI-ready datasets, including potential AI-related consequences of poor practice.

Protocol Element	What the Clinician Must Define Before Collection	Poor Practice	AI-Ready Practice	Potential AI Consequence
Patient eligibility	Inclusion criteria, exclusion criteria, care setting, and study period	Patients are added because their data are available	Patients are included according to predefined clinical criteria	Selection bias; limited generalizability
Unit of analysis	Whether one record represents a patient, admission, visit, image, lesion, procedure, signal segment, or clinical episode	Patients, admissions, images, and visits are mixed as if they were equivalent	One record type is clearly defined and consistently used	Non-independence; leakage; inflated sample size
Minimum common dataset	Core variables that must be collected for all eligible cases	Some patients have inflammatory markers, others have renal markers, others have only demographics	The same mandatory variables are collected for all eligible patients whenever possible	Inconsistent predictors; excessive missingness
Variable status	Which variables are mandatory, recommended, or optional	All available variables are added without deciding which are essential	Variables are classified before collection according to their role in the intended AI task	Unstable feature set; overfitting risk
Laboratory timing	Clinical time point of each laboratory test	CRP is taken from admission for some patients and from discharge for others	Separate variables are created, such as CRP_at_admission and CRP_at_discharge	Temporal leakage; invalid predictors
Imaging acquisition	Modality, acquisition date, anatomical region, protocol, file format, and link to patient/outcome	Image folders are collected without protocol, date, or patient-level linkage	Each image is linked to patient ID, acquisition metadata, label, and outcome	Poor reproducibility; linkage errors
Physiological signals	Signal type, device, sampling rate, duration, channels, acquisition conditions, and artifacts	ECG, EEG, or other signals are stored without technical parameters	Signals are stored with acquisition metadata and clear segmentation rules	Artifact bias; inconsistent inputs
Textual records	Type of document, source, date, anonymization status, and intended use	Notes and reports are copied as free text without structure or anonymization plan	Textual data are linked to patient records, dated, anonymized, and documented	Leakage; privacy risk; extraction inconsistency
Outcome or label	Outcome definition, confirmation source, measurement time point, and label type	Labels such as severe, complicated, or improved are used without criteria	Outcomes are defined using explicit clinical criteria and a documented source	Label ambiguity; misclassification
Missing values	Reason why a value is absent	Empty spreadsheet cells are left without explanation	Missingness is coded as not measured, not indicated, unavailable, not applicable, refused, lost to follow-up, or extraction error	Bias; inappropriate imputation/handling
Data source	Preferred source for each variable	Diagnoses, dates, or treatments are copied inconsistently from different documents	A preferred source is defined, such as specialist report, operative report, laboratory system, or pathology report	Provenance uncertainty; poor reproducibility
Consistency control	Person responsible for data entry and checking	Multiple people complete the dataset using different conventions	Data entry rules, coding conventions, and consistency checks are defined before collection	Systematic entry errors; reduced reliability

**Table 2:** Examples of minimum common dataset components for different clinical AI study types.

Study Type	Main AI Task	Minimum Common Dataset Should Include	Variables That Should Remain Optional or Exploratory
Diagnostic classification	Identify whether a patient has a condition	Patient identifier, age, sex, inclusion criteria, diagnostic reference standard, key predictors available before diagnosis, date of assessment	Rare biomarkers measured only in selected suspected cases
Prognostic prediction	Predict a future clinical event	Baseline patient data, comorbidities, predictors available at the prediction moment, clearly defined follow-up interval, event status	Variables measured after the outcome or only during late follow-up
Treatment-response modeling	Predict or classify response to therapy	Baseline status, treatment type, pre-treatment variables, response definition, response assessment time point	Post-response variables used as predictors for baseline response prediction
Laboratory-based ML study	Use laboratory markers for prediction or classification	Same mandatory laboratory markers for all eligible patients, units, collection time point, reference outcome	Additional tests performed only in severe or selected patients
Imaging-based AI study	Classify or predict using medical images	Patient identifier, imaging modality, acquisition date, anatomical region, protocol, label, outcome linkage	Images without acquisition metadata or without reliable label
Physiological signal study	Analyze ECG, EEG, EMG, spirometry, or sensor data	Signal type, device, sampling rate, recording duration, acquisition conditions, patient identifier, label	Signal segments without technical parameters or unclear timing
Longitudinal/follow-up study	Predict change, recurrence, survival, or complications over time	Baseline data, follow-up schedule, event definition, censoring rule, repeated measurement structure	Follow-up values collected at irregular or undocumented intervals
Multimodal study	Combine clinical, laboratory, imaging, signal, text, or follow-up data	Common patient identifier, synchronized time points, source of each modality, outcome, linkage rules	Modalities available only for small, biased, or undocumented subgroups

**Table 3:** Practical planning ranges for dataset size, target class balance, and model type in medical AI studies.

Dataset Situation	Tabular ML Interpretation	CNN/DL Imaging Interpretation	Class-Balance Warning
Fewer than 100 independent patients	Usually suitable only for descriptive analysis, feasibility assessment, or pilot exploration. Very fragile for ML, especially with many predictors.	Usually insufficient for CNN training. May support only proof-of-concept analysis or very limited transfer-learning exploration.	If the minority target class has fewer than 20 cases, reliable classification is usually not defensible.
100–300 independent patients	Exploratory tabular ML may be possible if the target is clear, predictors are few, NaN values are limited, and classes are reasonably balanced.	Small imaging pilot only. Transfer learning may be explored, but robust CNN claims are not justified.	If one class dominates, the limiting factor is the minority class, not the total number of patients.
300–1000 independent patients	More plausible for tabular ML if variables are clean, predictors are clinically justified, and enough target events exist. Still limited for broad generalization.	May support exploratory CNN or transfer-learning studies if images are well labeled, protocols are documented, and splitting is performed at patient level.	Each target class should ideally contain a clinically meaningful number of independent cases; very small classes remain unstable.
1000–5000 independent patients	Stronger basis for clinical ML, internal validation, subgroup checks, and more stable performance estimates.	More realistic for CNN/DL, especially when each class contains hundreds or thousands of independent patient-level cases.	Class imbalance must still be reported; a large total sample does not compensate for a very small minority class.
More than 5000 independent patients	More robust basis for ML, especially with multiple predictors, rare outcomes, and validation needs.	Better suited for DL, multicenter imaging, external validation, and more generalizable models.	Even large datasets require target-class counts, calibration assessment, and validation in clinically relevant subgroups.

**Table 4:** Clinical AI-readiness checklist for medical datasets.

Domain	Checklist Question	Complete	Partially Complete	Incomplete
Clinical task	Is the intended AI task clearly defined?	The task states what is predicted, classified, detected, or monitored, for whom, at what clinical moment, and for what intended use.	The task is generally defined, but the target population, timing, or intended use is incomplete.	The task is vague or only states that AI/ML will be applied.
Cohort definition	Are inclusion criteria, exclusion criteria, care setting, study period, and unit of analysis defined?	All cohort rules and the unit of analysis are documented before data extraction or collection.	Some cohort rules are defined, but the unit of analysis, time frame, or exclusion rules are unclear.	Patients or records are included only because data are available.
Minimum common dataset	Are mandatory variables collected consistently for eligible cases?	The same mandatory variables are collected for all or nearly all eligible cases.	Mandatory variables are mostly available, but relevant gaps remain.	Each patient has a different subset of variables, with no stable common core.
Outcome or label	Is the outcome clinically defined and linked to a confirmation source?	The outcome has explicit criteria, source, timing, and label type.	The outcome is defined, but confirmation source, timing, or label type is incomplete.	The outcome is vague, subjective, inconsistently assigned, or missing.
Measurement timing	Is the clinical time point of each variable documented?	Variables are linked to baseline, admission, diagnosis, treatment, discharge, or follow-up as appropriate.	Some variables have documented time points, but others remain unclear.	Values from different time points are mixed in the same column.
NaN and missing data	Are missing values coded and explained?	Missingness reasons are documented using predefined codes.	Missingness is partly coded, but many NaN values remain unexplained.	Empty cells or NaN values are left without explanation.
Standardization	Are units, formats, categories, and coding rules standardized?	Units, categorical values, dates, identifiers, and coding conventions are consistent.	Some variables are standardized, but others remain mixed or unclear.	Units, formats, or categories are inconsistent across records.
Non-tabular data	Are imaging, signal, text, pathology, or other files linked and documented?	Files are linked to patient ID, time point, metadata, label, and outcome.	Files are linked, but metadata, timing, or labeling information is incomplete.	Files are stored without reliable linkage, metadata, or label documentation.
Data dictionary	Is a complete data dictionary available?	Each variable has a name, definition, type, unit, source, time point, missing-data rule, and role in the study.	A data dictionary exists but is incomplete or not updated.	No data dictionary exists.
Validation readiness	Can the dataset support meaningful model evaluation?	Sample size, class balance, label reliability, leakage risk, patient-level splitting, and validation strategy are reviewed.	Some validation issues are known but not fully resolved.	The dataset cannot support a defensible validation plan.

**Table 5:** Illustrative application and reassessment of the Clinical AI-Readiness Checklist in a hypothetical clinical dataset.

Domain	Initial Score	Initial Assessment and Required Action	Reassessment Score
Clinical task	2	The prediction target, population, prediction moment, and intended use are explicitly defined. No corrective action is required.	2
Cohort definition	2	Inclusion and exclusion criteria, study period, care setting, and surgical episode as the unit of analysis are documented.	2
Minimum common dataset	1	Most mandatory variables are available, but selected laboratory variables remain unavailable for some eligible cases. These limitations are retained and documented.	1
Outcome or label	2	The 30-day postoperative complication has explicit clinical criteria, a documented confirmation source, and a fixed assessment window.	2
Measurement timing	0	Preoperative and postoperative CRP measurements are mixed within the same variable. Measurements are separated by clinical time point, and only values available before surgery remain eligible as predictors.	2
NaN and missing data	1	Missingness is only partly documented. Missing laboratory values are reviewed and assigned predefined missingness categories when the source information permits this.	2
Standardization	2	Laboratory units, categorical values, identifiers, and date formats are consistently standardized.	2
Non-tabular data	1	Imaging files are linked to the correct patients, but acquisition metadata are incomplete. Available modality, acquisition-date, and protocol metadata are completed.	2
Data dictionary	1	The data dictionary exists but does not completely define all variables, timing rules, and missing-data codes. It is updated accordingly.	2
Validation readiness	2	Patient-level independence, outcome distribution, leakage risk, and a feasible internal validation strategy have been reviewed.	2
Total	14/20		19/20

## Data Availability

No new data were created or analyzed in this study. Data sharing is not applicable to this article.

## Supplementary Materials

The following supporting information can be downloaded at: https://www.mdpi.com/article/10.3390/jcm15166297/s1, Table S1: Clinical AI-Readiness Checklist for Medical Datasets (fillable spreadsheet).
